# *TP53*-mutant CHIP defines a pro-inflammatory phenotype and predicts adverse outcomes after CAR T-cell therapy

**DOI:** 10.1038/s41375-026-03083-1

**Published:** 2026-08-24

**Authors:** Giulia Rappa, Frank Ziemann, Kieron White, Linus Kruk, Samar Shamas, Amelie Muth, Kayleen Shi, Cristina Zucchinetti, Savanna Süß, Maja Rothenberg-Thurley, Alessandra Holzem, Tobias Tix, Agnese Petrera, Bianka Ksienzyk, Charlotte Bentenrieder, Katharina S. Götze, Fabian Müller, Veit Bücklein, Kai Rejeski, Marion Subklewe

**Affiliations:** 1 Department of Medicine III, University Hospital, LMU Munich, Munich, Germany; 2 Laboratory for Translational Cancer Immunology, LMU Gene Center, Munich, Germany; 3 The Bavarian Cancer Research Center (BZKF), Munich, Germany; 4 Experimental Leukemia and Lymphoma Research (ELLF), LMU Munich, Munich, Germany; 5 Laboratory for Leukemia Diagnostics (LFL), LMU Munich, Munich, Germany; 6 German Cancer Consortium (DKTK) and German Cancer Research Center (DKFZ), Heidelberg, Germany; 7 Laboratory for Precision Immunotherapy, Department of Medicine III, University Hospital, LMU Munich, Munich, Germany; 8 Metabolomics and Proteomics Core Facility, Helmholtz Zentrum Munich—German Research Center for Environmental Health, Munich, Germany; 9 Department of Medicine III, Klinikum Rechts der Isar, Technical University of Munich (TUM) Hospital, Munich, Germany; 10 Department of Internal Medicine 5-Hematology and Oncology, University Hospital, Friedrich-Alexander-Universität Erlangen-Nürnberg, Erlangen, Germany

**Keywords:** Translational research, Immunotherapy

## Abstract

Clonal hematopoiesis of indeterminate potential (CHIP) has been well-characterized in patients receiving chemotherapy and hematopoietic cell transplantation. However, its immunologic relevance and potential role in modulating chimeric antigen receptor T-cell (CAR-T) therapy-related toxicities, inflammation, and clinical outcomes remains incompletely defined. In this exploratory study of 104 CAR-T recipients, we investigated the prognostic impact of pre-existing CHIP clones on toxicity and survival, and examined CHIP-associated inflammatory proteomic signatures leveraging longitudinal serum samples. Overall CHIP status was not associated with inflammatory toxicities, outcomes, or systemic inflammatory profiles. Analysis of clonal dynamics revealed *TP53-* and *ASXL1*-mutated clones to be the dominant expanding CHIP clones post-infusion, whereas *DNMT3A*- and *PPM1D*-mutated clones showed reduced clonal abundance over time. Notably, *TP53*-mutated CHIP was linked to lower platelet and hemoglobin levels, a higher baseline CAR-HEMATOTOX score, inferior clinical outcomes, and a distinct inflammatory signature. These findings identify *TP53*-mutated CHIP in CAR-T-treated patients as a high-risk CHIP genotype compared with other CHIP mutations. Importantly, patients who developed treatment-emergent myeloid neoplasms after CAR T-cell therapy exhibited distinct patterns of clonal expansion and inflammatory profiles. Together, these findings support a risk-adapted approach prioritizing longitudinal monitoring of *TP53*-mutated CHIP, especially in patients with high CAR-HEMATOTOX scores who develop cytopenias.

## Introduction

The development of chimeric antigen receptor T-cell (CAR-T) therapy has marked a major achievement in immunotherapy, significantly improving survival outcomes for a range of refractory B-cell malignancies [1–4]. Despite now representing a standard-of-care treatment, more than 50% of CAR-T recipients ultimately do not achieve a durable response. Furthermore, patients treated with CAR T cells often develop treatment-related toxicities such as cytokine release syndrome (CRS), immune effector cell-associated neurotoxicity syndrome (ICANS), and immune effector cell-associated hematotoxicity (ICAHT), which contribute to morbidity and mortality [5, 6]. A major contributor to CAR-T-associated toxicities is the significant inflammatory response, characterized by the profound release of pro-inflammatory cytokines upon target antigen recognition and subsequent CAR-T expansion [7].

Clonal hematopoiesis of indeterminate potential (CHIP) is characterized by somatic mutations in genes linked to myeloid malignancies, detected in blood or bone marrow at a variant allele frequency of ≥2%, in the absence of diagnostic features of a defined myeloid neoplasm [8]. In patients with large B-cell lymphoma (LBCL) and multiple myeloma (MM), the prevalence of CHIP ranges from 30 to 56% and has been associated with inferior survival outcomes and the development of myeloid neoplasm post cytotoxic-therapy (MN-pCT) following autologous hematopoietic stem-cell transplantation (auto-HCT) [9–12]. In this context, *TP53*-mutated CHIP emerged as the most prevalent mutation, highlighting its clinical relevance [13]. Notably, *TP53*-mutant clones have been linked to immunosuppressive tumor microenvironments that may influence treatment response [14]. Despite this, the role of *TP53-*mutant clones in the context of CAR-T remains unclear.

While a high prevalence of CHIP has been described in CAR-T recipients, the clinical sequelae of clonal hematopoiesis remain undefined, with prior studies producing heterogenous findings [9, 12, 15–19]. While some studies reported increased CRS/ICANS rates and effects on long-term outcomes [16–19], others did not reveal significant associations with CAR-T-related toxicities or efficacy (Table S1) [9, 12, 15]. Importantly, knowledge gaps persist regarding the specific inflammatory patterns associated with CHIP clones following CAR-T infusion, especially in the context of distinct CHIP mutations. Given the heterogeneity of prior findings, this study aims to elucidate the impact of CHIP on toxicities, survival, and inflammation after CD19- or BCMA-directed CAR-T therapy.

## Materials and methods

### Patients and data collection

In this observational study, we investigated toxicity and treatment outcomes in 104 patients treated with standard-of-care Axi-cel (*n* = 46), Tisa-cel (*n* = 16), Brexu-cel (*n* = 13), Liso-cel (*n* = 10), Ide-cel (*n* = 15), and Cilta-cel (*n* = 4) for r/r NHL or MM. Patients were treated between 01/2019 and 03/2025 at LMU University Hospital. Lymphodepleting chemotherapy using fludarabine and cyclophosphamide was administered according to the manufacturer’s guidelines. Clinical metadata were obtained from electronic medical records with institutional review board approval (LMU Nr. 19-817).

### Toxicity grading and clinical outcomes

CRS and ICANS were graded according to ASTCT consensus criteria [20]. Early and late ICAHT were graded using the EHA/EBMT classification system [21, 22]. Management of treatment-related toxicity and antimicrobial prophylaxis adhered to local institutional guidelines. Efficacy assessment followed Lugano and IMWG criteria. Progression-free survival (PFS) and overall survival (OS) were analyzed using the Kaplan-Meier estimates from date of infusion, and groups were compared by log-rank test. Non-relapse mortality (NRM) was defined as death unrelated to relapse or progression and compared between groups using Gray’s test in a competing-events framework.

### CHIP detection

Peripheral blood mononuclear cells (PBMC) were collected around lymphodepletion, as well as month 1, 3, 6, and 12 post-infusion; 9/26 patients with CHIP-associated mutations relapsed within the first 6 months post CAR-T infusion. After month 6, follow-up samples were available for 10/17 patients. Genomic DNA was extracted from PBMCs and analyzed using a targeted sequencing panel covering 74 genes recurrently mutated in myeloid and lymphoid disorders, as outlined in Table S2. Sequencing was performed using the Haloplex platform (Agilent, Germany) and MiSeq technology (Illumina, San Diego, USA). Reads were aligned using the Burrows-Wheeler Aligner, and variants were detected with VarScan2 and Pindel, as previously described [23]. Only variants with a VAF of at least 1% were included in downstream analyses [12, 24, 25].

### Serum proteomic analysis

Longitudinal serum proteomics profiling was performed using prospectively collected serum samples from 83 patients at pre-defined time points (days 0, 2–7, 14, 28, 90, 180, 360) using the 92-protein multiplex proximity extension assay from the Olink^®^ Immuno Oncology Panel (see Supplementary Methods for further details) [26].

### Statistical analysis

We applied the Wilcoxon rank-sum test to analyze continuous data. Categorical variables were examined using Fisher’s exact test. Receiver operating characteristic (ROC) curve analysis explored the discriminative ability of baseline clinical variables for the presence of *TP53*-mutated CHIP. Following variable selection, a simplified baseline risk score was derived by assigning one point for CAR-HEMATOTOX (HT) score ≥2 and one point for age >65 years. A Jonckheere-Terpstra (J-T) trend test was used to explore white blood cell (WBC) count recovery. All statistical analyses and data visualisation were conducted using GraphPad Prism (v11.0.0), Olink Insight-Stat Analyzer, and RStudio (v26.4). Two-sided *p* values < 0.05 were considered statistically significant.

## Results

### CHIP is associated with age and more common in patients with multiple myeloma

**Fig. 1:**
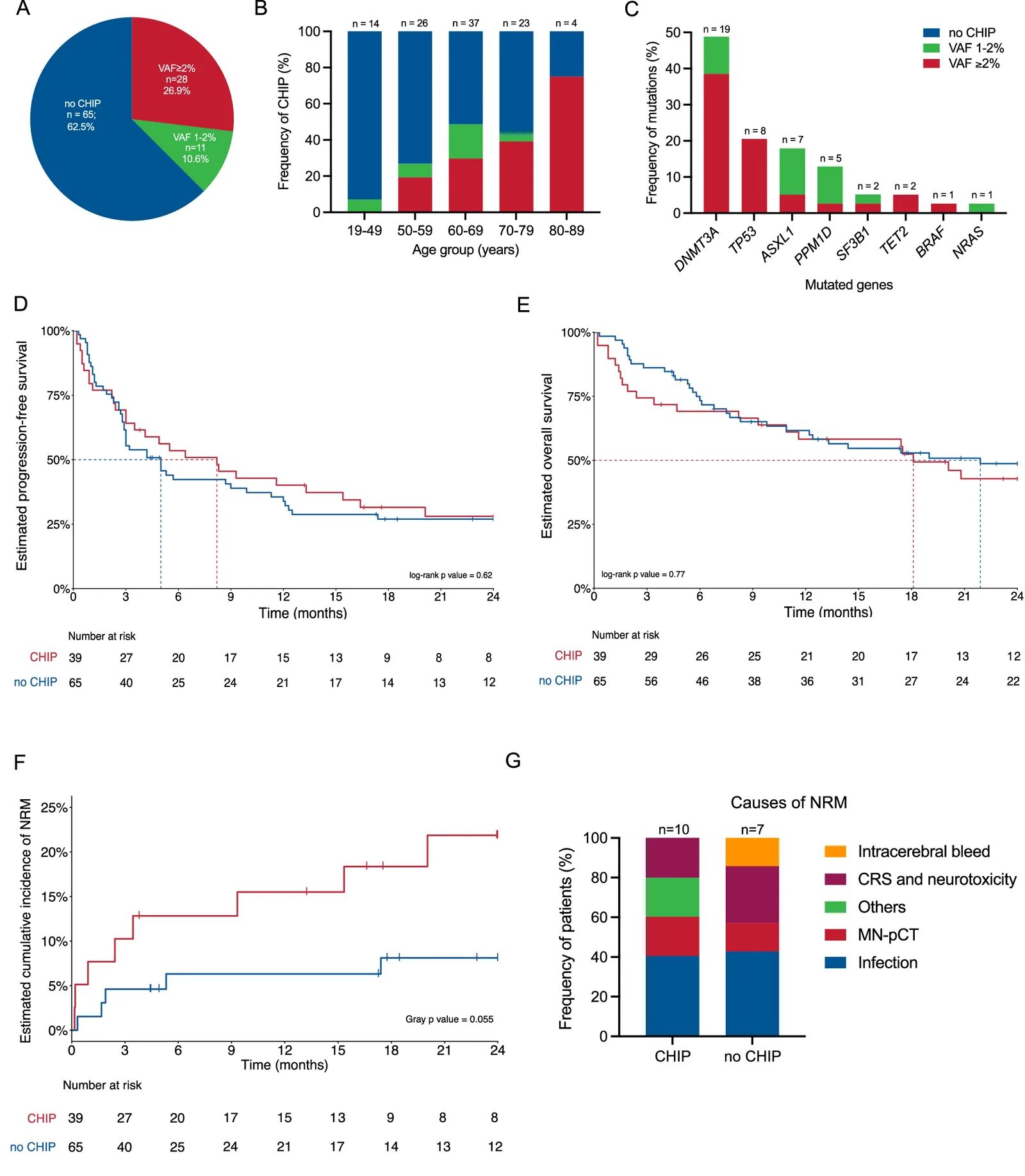
CHIP mutations frequency increases with age and elevates NRM risk but does not affect patients’ survival after CAR T-cell therapy. **A** Occurrence of CHIP among 104 patients receiving CD19 or BCMA CAR T-cells. **B** Prevalence of CHIP across age group. **C** Frequency of mutations among CHIP patients. **D** Progression free survival and **E** overall survival of patients with CHIP (*n* = 39) or without CHIP (*n* = 65). **F** Cumulative incidence of NRM in CHIP (*n* = 39) versus without CHIP (*n* = 65) patients. **G** Causes of NRM in patients with CHIP (left, *n* = 10) or without CHIP (right, *n* = 7). PFS and OS were compared using the Kaplan-Meier estimates and the log-rank test; hazard ratios were estimated using Cox proportional hazards model. NRM was analyzed using cumulative incidence functions and groups were compared using Gray’s test.

Patients received CAR-T therapy after a median of three prior lines of treatment. Median follow-up was 34.2 months. We identified CHIP-related mutations in 39/104 patients (VAF ≥ 1%: 11/39; VAF ≥ 2%: 28/39) prior to infusion (Fig. 1A). As expected, the CHIP group was significantly older than the group without CHIP mutations (median 66 vs. 60 years, *p* = 0.002). Accordingly, we observed an increase in CHIP prevalence across age groups, starting from 7% in the youngest group (age: 19-49, VAF ≥ 1), to 27% in the 50–59 age group, followed by 49% in the 60-69 age cohort, 43% in the 70–79 age group and 75% in the oldest group (age: 80–89; Table 1, Fig. 1B). The most frequently mutated gene was *DNMT3A* (*n* = 19), followed by *TP53* (*n* = 8), *ASXL1* (*n* = 7), *PPM1D* (*n* = 5), *TET2* and *SF3B1* (each *n* = 2), and *NRAS* and *BRAF* (each *n* = 1; Fig. 1C). Sex distribution was similar across cohorts with CHIP (female: 46%, male: 54%) and without CHIP (female: 37%, male: 63%) (Table 1, *p* = 0.41). A higher proportion of MM patients displayed baseline CHIP clones (10/19, 52.6%) compared with the B-NHL cohort (29/85, 34.1%), although this did not reach statistical significance (*p* = 0.19). In terms of prior treatment lines, most patients (96%) were previously exposed to an alkylating agent, regardless of CHIP status. Autologous hematopoietic cell transplantation was more commonly administered in the CHIP cohort (41% vs. 31%, Table 1). We observed a trend toward a longer interval from initial diagnosis to infusion in the CHIP cohort versus patients without CHIP (48.3 vs. 19.9 months [mo], *p* = 0.11).

**Table 1:** Patient demographics, clinical characteristics, and laboratory parameters.

	All patients (*N* = 104)^a^	CHIP (*n* = 39)^a^	No CHIP (*n* = 65)^1^	*p* value
**Clinical characteristics**				
**Age, years**	64 [56; 70]	66 [62; 73]	60 [53; 68]	**0.002b**
**Sex**				0.411c
female	42 (40%)	18 (46%)	24 (37%)	
male	62 (60%)	21 (54%)	41 (63%)	
**Diagnosis**				0.189c
B-NHL	85 (82%)	29 (74%)	56 (86%)	
MM	19 (18%)	10 (26%)	9 (14%)	
**CAR-T product**				0.673c
Axi-cel	46 (44%)	16 (41%)	30 (46%)	
Liso-cel	10 (9.6%)	4 (10%)	6 (9.2%)	
Cilta-cel	4 (3.8%)	2 (5.1%)	2 (3.1%)	
Tisa-cel	16 (15%)	6 (15%)	10 (15%)	
Brexu-cel	13 (13%)	3 (7.7%)	10 (15%)	
Ide-cel	15 (14%)	8 (21%)	7 (11%)	
**Prior lines of therapy**	3 [2; 4]	3 [2; 5]	3 [2; 4]	0.239b
**Prior auto-HCT**	36 (35%)	16 (41%)	20 (31%)	0.297c
**Prior exposure to alkylating agents**	100 (96%)	37 (95%)	63 (97%)	0.630^c^
**Time from initial diagnosis to CAR-T, months**	26.9 [13.6; 71.5]	48.3 [15.6; 76.5]	19.9 [12.3; 59.2]	0.109^b^
**CAR-Hematotox score**	1 [0; 3]	1 [0; 3]	1 [0; 3]	0.997^b^
**Laboratory parameters at time of lymphodepletion**				
**ANC x 10**^**9**^**/L**	2.42 [1.50; 3.70]	2.45 [1.62; 3.57]	2.38 [1.44; 3.82]	0.832^b^
**Platelets x 10**^**9**^**/L**	177 [117; 256]	176 [90; 276]	177 [134; 254]	0.680^b^
**Hemoglobin g/dL**	10.10 [8.70; 11.45]	10.00 [8.70; 11.30]	10.10 [8.70; 11.60]	0.859^b^
**CRP mg/dL**	0.65 [0.20; 2.75]	0.80 [0.20; 2.70]	0.60 [0.20; 2.80]	0.877^b^
**Ferritin ng/mL**	374 [121; 1,062]	374 [107; 1,062]	374 [145; 843]	0.952^b^
**LDH U/L**	241 [186; 365]	241 [188; 383]	240 [180; 336]	0.511^b^

*CHIP* clonal hematopoiesis of indeterminate potential, *B-NHL* B-cell Non-Hodgkin lymphoma, *MM* multiple myeloma, *Axi-cel* Axicabtagene ciloleucel, *Liso-cel* Lisocabtagene maraleucel, *Cilta-cel* Ciltacabtagene autoleucel, *Tisa-cel* Tisagenlecleucel, *Brexu-cel* Brexucabtagene autoleucel, *Ide-cel* Idecabtagene vicleucel, *Auto-HCT* autologous hematopoietic cell transplantation, *ANC* absolute neutrophil count, *CRP* C-reactive protein, *LDH* lactate dehydrogenase.

^a^Median [Q1; Q3]; n (%).

^b^Wilcoxon rank-sum test.

^c^Fisher’s exact test.

### Overall CHIP status did not associate with CAR-T toxicities or clinical outcomes

When studying the influence of CHIP on CAR-T-mediated toxicities, we did not detect significant differences between patients with and without CHIP. In particular, no differences in CRS, ICANS, early and late ICAHT, or neutrophil recovery phenotypes were detected (Table 2). However, a trend towards higher 2-year cumulative NRM incidence was found in the CHIP group compared to the group without CHIP (21.9% vs 8.1%, *p* = 0.055; Fig. 1F). In the CHIP cohort, the primary cause of NRM was infection (4/10), followed by CAR-T-mediated toxicities (2/10), clinical deterioration (2/10), and MN-pCT (2/10). In the group without CHIP, infection also represented the leading cause of NRM (3/7), followed by CAR-T-related toxicities (2/7) intracerebral bleed, and MN-pCT (1/7 each, Fig. 1G).

**Table 2:** CHIP in the context of CAR T-cell-mediated toxicities.

	All patients (*N* = 104)^a^	CHIP (*n* = 39)^a^	No CHIP (*n* = 65)^a^	*p* value^b^
**CRS (all grades)**	94	38	56	0.086
**CRS (G** ≥ **3)**	14	5	9	>0.999
**ICANS (all grades)**	36	14	22	0.835
**ICANS (G** ≥ **3)**	13	5	8	>0.999
**Early ICAHT**				
All grades	94	33	61	0.657
Severe (G ≥ 3)	22	8	14	0.805
NA	5	5	0	
**Late ICAHT**				
All grades	67	23	44	0.734
Severe (G ≥ 3)	32	9	23	0.338
NA	27	12	15	
**ANC phenotype**				
quick	25	9	16	>0.999
intermittent	58	21	37	>0.999
aplastic	18	7	11	>0.999
NA	3	2	1	

*CRS* cytokine release syndrome, *ICANS* immune effector cell-associated neurotoxicity syndrome, *ICAHT* immune effector cell-associated hematotoxicity, *ANC* absolute neutrophil count.

^a^n.

^b^Fisher’s exact test.

Assessment of CAR-T outcomes (Fig. 1D, E) revealed no significant differences in PFS and OS for patients with and without CHIP (median PFS: 8.2 vs 5.0 mo, *p* = 0.62; median OS: 18.1 vs 21.9 mo, *p* = 0.77). Stratification by disease entity did not reveal significant survival differences for LBCL and MM (Fig. S1). However, diminished survival was observed for MCL patients harboring CHIP mutations (CHIP vs no CHIP: median PFS, 2.4 vs 17.2 mo, *p* = 0.12; median OS, 2.4 vs 25.6 mo, *p* = 0.04, Fig. S1E and S1F), a finding that was recapitulated in the Brexu-cel subgroup (Fig. S2G and S2H).

### Inflammatory and cytopenia profiles were comparable by CHIP status

To characterize the inflammatory status of patients harboring CHIP mutations, we analyzed standard laboratory values and performed serum proteomic profiling using the Olink platform. No significant differences were observed in baseline CRP and ferritin levels between both groups (CHIP vs no CHIP, CRP: 0.8 vs 0.6 mg/dL, *p* = 0.88; Ferritin: 374 vs 374 ng/mL, *p* > 0.9; Table 1). OLINK analysis did not reveal substantial differences in serum proteomic profiles between the cohorts with and without CHIP. Merely one single protein, CD8A, was significantly upregulated in the group without CHIP (adj *p* = 0.047, Fig. S3). Examining baseline cytopenia, we observed no difference in platelet, hemoglobin, and absolute neutrophil counts between the two cohorts (Table 1). In 71 patients with evaluable flow cytometric CAR-T expansion data (Fig. S4), we did not find significant differences (median area under the curve [AUC] CHIP vs. no CHIP: 577 vs 363 cells/μL, *p* = 0.84).

### CHIP clones exhibit distinct temporal dynamics

**Fig. 2:**
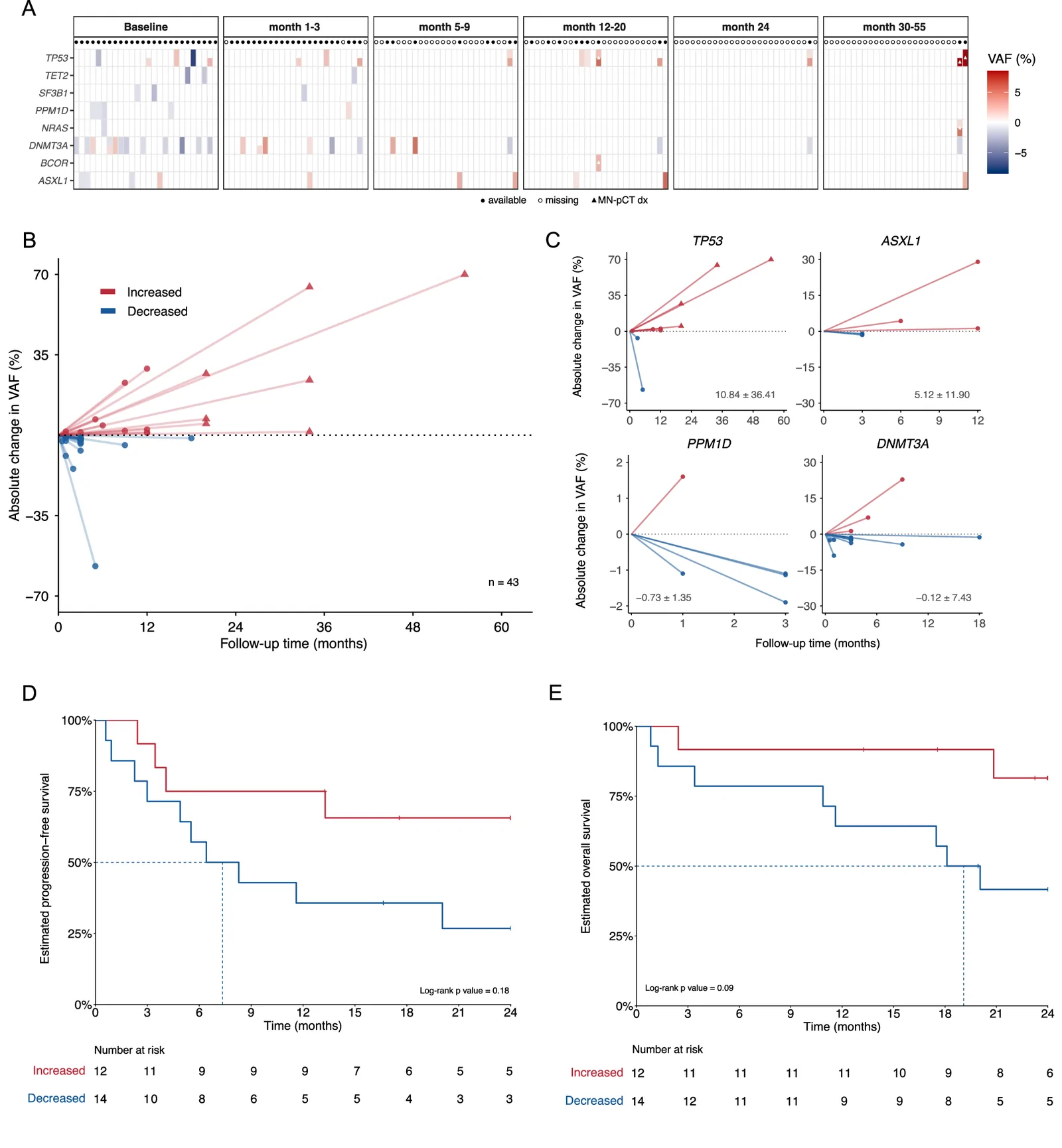
Longitudinal analysis reveals a distinct dynamic of CHIP clonal expansion. **A** NGS-based mutation landscape in PBMC cells from CHIP patients at baseline (*n* = 26), month 1–3, month 5–9, month 12–20, month 24, and month 30–55. Red indicates an increase in VAF percentage while blue indicates a reduction in VAF percentage. Split boxes indicate different mutations on the same gene. Open dots denote missing samples, whereas closed dots indicate available samples at the given timepoint. Triangles mark the time point at which the MN-pCT diagnosis occurred. **B** Absolute change in VAF between month 0 and 60 for all 43 clones. Red and blue lines describe increasing and decreasing VAFs respectively. The absolute change in VAF was calculated as the difference between baseline (day 0) VAF and the VAF at the timepoint of maximal expansion or maximal contraction. **C** Absolute change in VAF stratified per gene. Red and blue lines describe increasing and decreasing absolute VAFs, respectively. **D** Progression free survival and **E** overall survival of patients characterized by increased (red, *n* = 12) or decreased (blue, *n* = 14) CHIP clones. Patients were classified into the expansion group when the VAF at follow-up was higher than at baseline and when new mutations occurred. PFS and OS were compared using the Kaplan-Meier estimates and the log-rank test; hazard ratios were estimated using Cox proportional hazards model.

To investigate the clonal expansion of mutated clones over time, we longitudinally sequenced PBMC samples. 26 out of 39 patients had available follow-up samples, with timepoints varying from month 1 to 55. We detected 18 clones with a higher and 25 clones with a lower absolute VAF change over time (median absolute VAF change increase vs decrease: 4.6 vs 1.8, Fig. 2A, B). The individual clonal dynamics of patients with at least two mutations are depicted in Fig. S5. To further dissect whether certain CHIP clones display distinct dynamics, we examined the abundance of the different mutated clones over time and found that *TP53-* and *ASXL1-*mutated clones tend to expand more than clones with *PPM1D* and *DNMT3A* mutations. Specifically, 8/10 *TP53-*mutated patients and 3/6 *ASXL1*-mutated patients showed an increase in absolute VAF change. In contrast, *PPM1D*-positive patients exhibited a decrease in absolute VAF change in 4/5 cases and *DNMT3A* in 11/14 cases (Fig. 2C). To exclude peripheral blood involvement, multiparameter flow cytometry was performed before leukapheresis and after CAR-T infusion and revealed no circulating lymphoma or myeloma cells. Genetic analysis of tumors and bone marrow biopsies showed no overlap with CHIP mutations; notably, *TP53*^*mut*^ CHIP patients had no *TP53* tumor mutations.

Furthermore, we investigated whether the expansion of CHIP clones affected patient outcomes. In terms of hematotoxicity, both patients with and without CHIP expansion experienced a monotonic WBC decline between day –5 to 7 (*p*_JT_ < 0.001, respectively, Table S3). However, WBC recovery during day 7–30 showed a positive trend only in patients without CHIP expansion (*p*_JT_<0.001). Interestingly, patients with clonal expansion showed numerically improved PFS and OS (Fig. 2D, E) compared with those without clonal expansion (median PFS: 34.9 vs 7.4 mo, *p* = 0.18; median OS: 60.2 vs 19.1 mo, *p* = 0.09).

### *TP53*-mutant CHIP is correlated with pancytopenia and a distinct inflammatory profile

**Fig. 3:**
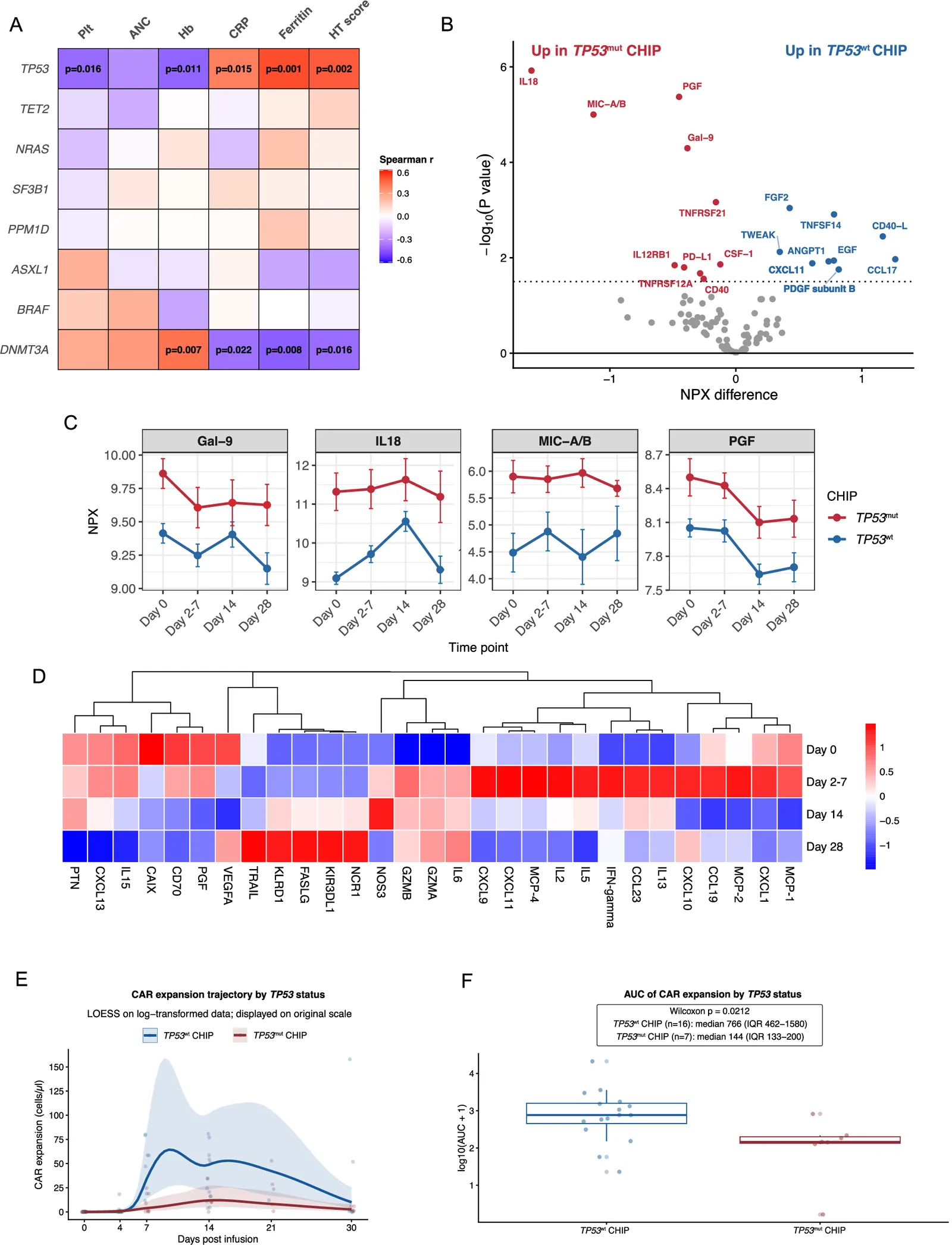
CHIP subgroup analysis demonstrates an association between *TP53*^mut^ CHIP, cytopenias, and inflammation. **A** Spearman rank correlation between peripheral blood counts and different CHIP mutations. **B** Volcano plot showing the estimated mean difference between *TP53*^*mut*^ CHIP and all other *TP53*^*wt*^ CHIP patients (x axis) and corresponding -log10 (y axis, adjusted *p* value) between day 0 and day 28. Red (*TP53*^*mut*^ CHIP) and blue (*TP53*^*wt*^ CHIP) indicate proteins that passed the multiple testing adjusted *p* value threshold of *p* < 0.05. **C** Top candidate proteins of interest related to *TP53*^*mut*^ CHIP patients. **D** Timepoint analysis showing the inflammatory signature of *TP53*^*mut*^ CHIP patients at day 0, 2 to 7, 14, and 28. Heatmap color scale indicates relative protein concentration; red denotes higher and blue denotes lower relative expression. **E** CAR-T expansion trajectory stratified by *TP53* status. **F** AUC of CAR T-cell-expansion by *TP53* CHIP status. In red *TP53*^*mut*^ CHIP (*n* = 7) and in blue *TP53*^*wt*^ CHIP (*n* = 16) cohorts.

To determine whether different CHIP mutations exhibit distinct characteristics, we next conducted a mutation-specific subgroup analysis. Evaluation of biological parameters at lymphodepletion (Fig. 3A, Table S4) showed that *TP53*-mutated CHIP (*n* = 8) was associated with baseline thrombocytopenia (median platelet count: *TP53*^*mut*^ versus *TP53*^*wt*^ CHIP: 91 vs 207 e9/L, *p* = 0.02) and anemia (median hemoglobin: *TP53*^*mut*^ versus *TP53*^*wt*^ CHIP: 8.85 vs 10.3 g/dL, *p* = 0.01). In addition, *TP53*^*mut*^ CHIP was linked to elevated baseline levels of CRP and ferritin (*TP53*^*mut*^ versus *TP53*^*wt*^ CHIP, median CRP: 2.3 vs 0.3 mg/dL, *p* = 0.02; median ferritin: 1256 vs 236 ng/mL, *p* = 0.001). Accordingly, the HT score was elevated in patients harboring *TP53* mutations (median HT score: *TP53*^*mut*^ versus *TP53*^*wt*^ CHIP: 3 vs 1, *p* = 0.003). Interestingly, *DNMT3A-*mutated clones exhibited opposite characteristics to *TP53-*mutated CHIP clones. Accordingly, *DNMT3A*-mutated patients showed significantly higher hemoglobin levels (median hemoglobin: *DNMT3A*^*mut*^ vs others: 11.1 vs 9.3 g/dL, *p* = 0.008) and lower CRP, ferritin and CAR-HEMATOTOX score before lymphodepletion (median *DNMT3A*^*mut*^ vs others, median CRP: 0.3 vs 2.1 mg/dL, *p* = 0.02, median ferritin: 207 vs 703 ng/mL, *p* = 0.009, median HT score: 1 vs 3, *p* = 0.02).

Given the correlation between *TP53*^*mut*^ CHIP, inflammatory patterns, and cytopenias, we further investigated the proteomic landscape of patients harboring *TP53*^*mut*^ CHIP (*n* = 7) with those carrying other CHIP mutations (*n* = 23). Interestingly, the *TP53*^*mut*^ CHIP group displayed a distinct proteomic signature (Fig. 3B and Fig. S6A) marked by upregulation of the inflammatory markers IL-18, PGF, IL12RB1, CD40, and tumor necrosis factors TNFRSF21, TNFRSF12A. In addition, the immune checkpoint ligands PD-L1, Gal-9, the growth factor CSF-1, and the stress factor MIC-A/B were found to be elevated in the *TP53*^*mut*^ CHIP cohort compared to the *TP53*^*wt*^ CHIP group. In contrast, the *TP53*^*wt*^ CHIP group was characterized by increased levels of the growth factors FGF2, EGF, ANGPT1, and PDGF subunit B and the tumor necrosis factors TWEAK and TNFSF14. In addition, the chemoattractant CXCL11 and CXCL17 and the inflammatory marker CD40L were found to be highly concentrated in the *TP53*^*wt*^ CHIP group compared to the *TP53*^*mut*^ CHIP cohort (Fig. 3B). Longitudinal assessment of the most abundant cytokines (IL-18, MIC-A/B, PGF, and Gal-9) demonstrated a significant and continuous increase across all timepoints between day 0 and day 28 (Fig. 3C).

Next, a timepoint-specific analysis of inflammatory pathways was performed separately for the *TP53*^*mut*^ group and the *TP53*^*wt*^ CHIP cohort. Tumor-intrinsic and lymphodepletion-associated markers such as VEGFA, PGF, CD70, CAIX, IL-15, CXCL13, and PTN were upregulated for both groups at day 0. Between day 2 and day 7, concentrations of activation and chemoattractant cytokines including MCP1, CXCL10, IFNy, IL-2, CXCL11, CXCL9, and IL-6 were similarly elevated in both cohorts. Interestingly, at day 28, a prolonged inflammatory state characterized by elevations of IL-6, GZMA and GZMB, CXCL10, and VEGFA characterized *TP53*^*mut*^ CHIP patients (Fig. 3D, Fig. S6B).

Both PFS and OS were diminished in patients harboring a *TP53* mutation compared to CHIP patients without *TP53* mutation, although this comparison was underpowered and did not reach statistical significance (median PFS: 2.9 vs 9.3 mo, *p* = 0.22; median OS *TP53*^*mut*^ CHIP vs *TP53*^*wt*^ CHIP: 9.9 vs 20.1 mo, *p* = 0.08, Fig. S6C and S6D). Of interest, CAR-T expansion was markedly reduced in patients harboring *TP53*^*mut*^ CHIP compared to *TP53*^*wt*^ CHIP (median AUC: 144 versus 766 cells/μL, Fig. 3E, F, *p* = 0.02).

### Advanced age and high CAR-HEMATOTOX score risk-stratify for *TP53*-mutant CHIP

**Fig. 4:**
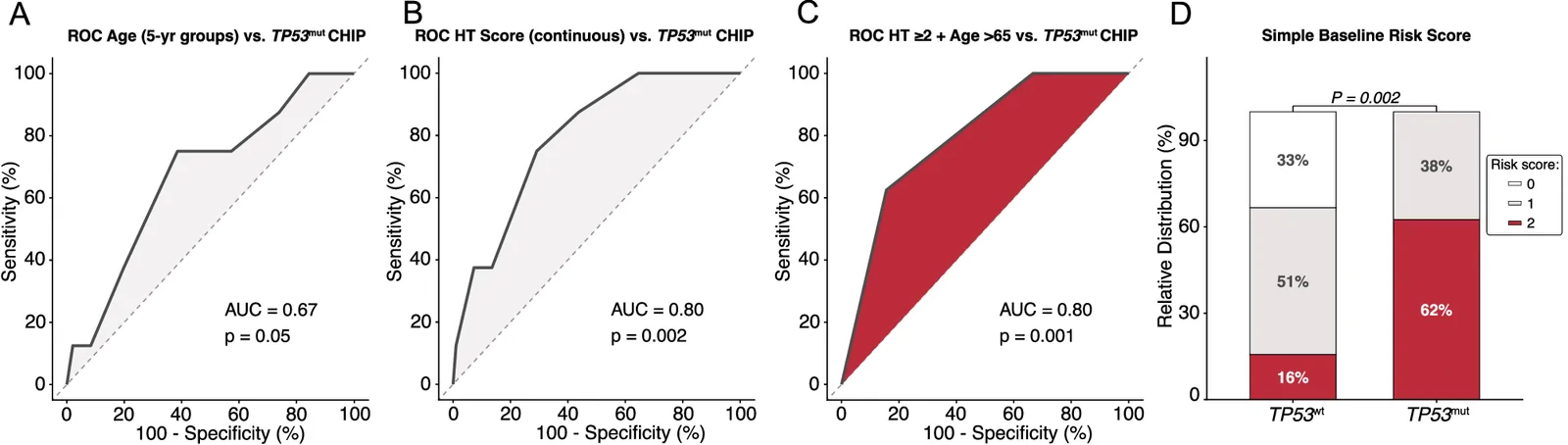
Advanced age and high CAR-HEMATOTOX score discriminate for *TP53* mutant CHIP. **A** ROC curve of age for *TP53*^*mut*^ CHIP discrimination. **B** ROC curve of HT score ( ≥ 2) for *TP53*^*mut*^ CHIP discrimination. **C** ROC curve combining age and HT for *TP53*^*mut*^ CHIP discrimination. The x-axis shows 100 - specificity (%) and the y-axis reports sensitivity (%). **D** Relative distribution of age and HT derived risk score among *TP53*^*mut*^ and *TP53*^*wt*^ CHIP patients. One risk point was assigned for each variable (age and HT).

Considering that age and the baseline HT score were associated with *TP53*^*mut*^ CHIP status, we next evaluated their discriminatory performance using ROC analysis. With an AUC of 0.67, age provided moderate discrimination for *TP53*^*mut*^ CHIP status (*p* = 0.05, Fig. 4A). In contrast, the HT score demonstrated improved discriminatory capacity, with an AUC of 0.80 (*p* = 0.002; Fig. 4B). To enhance specificity and facilitate clinical implementation, age >65 years and a HT score ≥2 were combined (1 point each) into a parsimonious risk score for enrichment of *TP53*^*mut*^ CHIP. Notably, the score achieved an AUC of 0.80 with improved test characteristics compared with age alone (score of 2: sensitivity 62.5%; specificity 84.4%; *p* = 0.001, Fig. 4C and Table S5). In the *TP53*^*mut*^ CHIP group, 62% (5/8) of patients had a score of 2 compared to only 16% (15/96) of patients in the *TP53*^*wt*^ CHIP group (*p* = 0.002; Fig. 4D, Table S6). At the threshold of 2, the score resulted in a number needed to test of 4 patients to identify one patient with *TP53*^*mut*^ CHIP. Together, these findings support the score’s potential utility for baseline risk stratification.

### MN-pCT patients were characterized by distinct *TP53*-mutant clonal expansion and inflammatory profiles

Among our cohort, three individuals receiving CAR T cells subsequently developed treatment-associated myeloid neoplasms bearing *TP53* mutations. The first 64-year-old patient was diagnosed with myelodysplastic syndrome (MDS-pCT, #1), while the second 72-year-old patient and third 68-year-old patient developed acute myeloid leukemia (AML-pCT, #2 and #3). Patients #1 and #2 were treated with Tisa-cel, while Patient #3 received Axi-cel. The onset of MN-pCT occurred 4.6, 2.8, and 1.7 years following CAR-T infusion, respectively. Generally, the subsequent outcome of the MN-pCT patients was poor with an OS ranging between 0.7 and 6.5 months. Two of the three patients were not able to receive further systemic therapy due to cytopenia and poor performance status. Detailed treatment history and post-CAR-T outcomes are provided in Fig. S7.

**Fig. 5:**
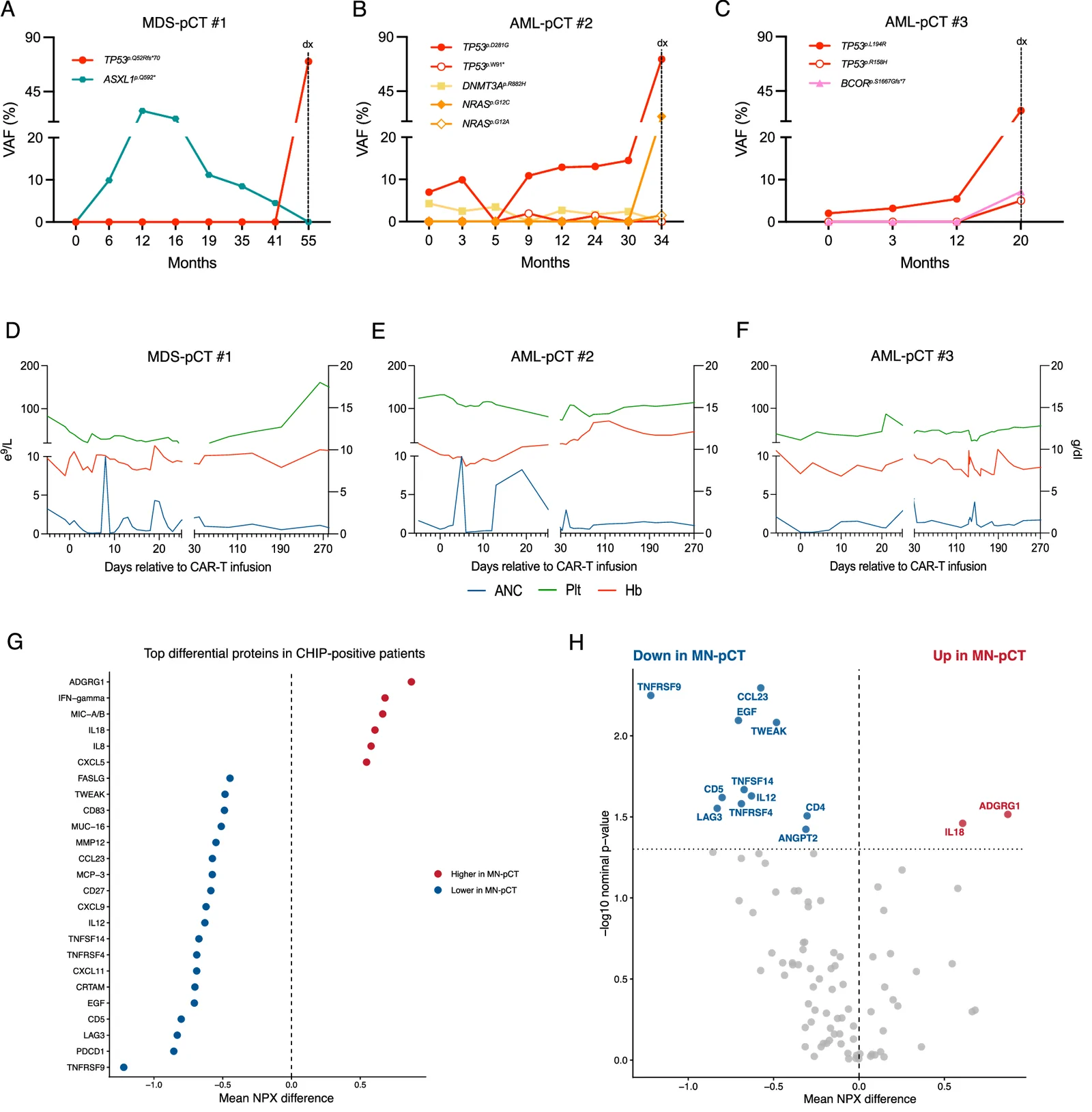
MN-pCT patients show distinct clonal expansion, cytopenias, and inflammatory signatures. **A**–**C** Longitudinal analysis of VAF dynamics in MDS-pCT #1, AML-pCT #2, AML-pCT #3 patients respectively. **D**–**F** Longitudinal plots describing MN-pCT cytopenias during the treatment with CAR T cells. The blue line depicts absolute neutrophil counts, the red line hemoglobin counts and the green line platelet counts. The left y-axis corresponds to ANC and Plt counts, the right y-axis represents hemoglobin counts. **G** Differential protein expression of the 25 proteins differing between CHIP patients without MN-pCT development (left) and MN-pCT patients (right). The x-axis represents the estimated NPX difference between the two groups. **H** Volcano plot showing the estimated mean difference between MN-pCT patients and all other CHIP patients (x-axis) and corresponding -log10 (y-axis, adjusted *p* value) between day 0 and day 360. Red (up in MN-pCT patients) and blue (down in MN-pCT) indicate proteins that passed the multiple testing adjusted *p* value threshold of *p* < 0.05.

Patient #1 was characterized by expansion of a *ASXL1*^p.Q592*^ clone peaking at month 12 (VAF: 29%), followed by a gradual contraction phase and then the occurrence of a *TP53*^p.G52Rfs*70^ mutation (VAF: 70%) at the time of MDS-pCT diagnosis (Fig. 5A). Patient #2 (Fig. 5B) exhibited contraction of a *DNMT3A*^*p.R882H*^ clone, a marked expansion of an initial *TP53*^*p.D281G*^ clone (VAF: 71.5%), occurrence of a *TP53*^p.W91*^ clone at month 9 and 24 (VAF: 1.9 and 1.4%, respectively) and presentation of two *NRAS* clones at the AML-pCT diagnosis (NRAS^p.G12C^ VAF: 24%; NRAS^p.G12A^ VAF: 1.5%). Patient #3 showed expansion of a *TP53*^*p.L194R*^ clone (VAF: 28.8%) with occurrence of an additional *TP53*^*p.R158H*^ and of *BCOR*^p.S1667Gfs*7^ clones (VAF: 5 and 7.1%, respectively) at the time of MN-pCT diagnosis (Fig. 5C). When examining the antecedent toxicity profile, the MDS-pCT patient exhibited grade 1 CRS, absence of ICANS, and grade 2 early and late ICAHT. Among the patients diagnosed with AML-pCT, patient #2 demonstrated grade 1 CRS and ICANS along with grade 2 and 3 early and late ICAHT, while patient #3 showed grade 1 CRS, absence of ICANS, and grade 2 early ICAHT (Fig. S8A).

Longitudinal assessment of hematologic parameters demonstrated consistent profound neutropenia across the entire CAR-T treatment period. However, the three patients with MN-pCT showed no prolonged anemia and thrombocytopenia (Fig. 5D–F). CRP levels were markedly elevated, reaching a peak between day -1 and day 4 in the MDS-pCT #1 and AML-pCT #3 patients and between day 3 and day 7 post-infusion in the AML-pCT #2 patient. Ferritin levels remained substantially elevated throughout the infusion period in all the three individuals (Fig. S8B,  S8C, and  S8D).

Analysis of the inflammatory milieu in patients who developed MN-pCT identified IL-18, ADGRG1, IFNγ, MIC-A/B, IL-8, and CXCL5 as the top upregulated proteins compared with CHIP patients who did not develop secondary malignancies (Fig. 5G); among these, IL-18 and ADGRG1 were significantly elevated (Fig. 5H). In contrast, TNFRSF9, CCL23, EGF, TWEAK, TNFSF14, CD5, IL-12, LAG3, TNFRSF4, CD4, and ANGPT2 were significantly downregulated in patients with MN-pCT compared to the cohort without MN-pCT development (Fig. 5G, H).

## Discussion

In this observational study, we investigated if CHIP influences clinical outcomes and immune-related toxicities in the setting of CAR-T treatment. We did not identify an association between overall CHIP status and CAR-T outcomes or inflammatory patterns. However, *TP53*^*mut*^ CHIP was linked to baseline cytopenias, high CAR-HEMATOTOX scores, a distinct inflammatory signature, and diminished CAR T-cell expansion.

To date, data outlining CHIP prevalence and associations between CAR-T therapy and clinical outcomes have been largely heterogenous (Table S1). In our analysis, we noted a prevalence of pre-existing CHIP of 37.5%, which was numerically increased in the myeloma sub-cohort (53% vs 34%) and similar in range to previous reports (Table S1). Consistent with previous studies, the frequency of CHIP increased with advancing age groups and the mutational spectrum shifted towards DNA damage repair genes [9, 16]. Overall, *DNMT3A*, *TP53*, and *ASXL1* were the most frequently mutated genes. In line with prior findings [9, 12], we found no correlation between overall CHIP status and major toxicities. Furthermore, CHIP status did not translate into differences in survival [9, 12, 18]. Despite this, CAR-T recipients harboring CHIP mutations showed a tendency towards increased NRM.

Previous studies have demonstrated that exposure to chemotherapy exerts selective pressure favoring clones carrying mutations in DNA damage repair genes (e.g., *TP53*, *PPM1D*), whereas alterations in epigenetic regulators (e.g., *DNMT3A, TET2, ASXL1*) are predominantly age-related and typically not enriched following chemotherapeutic exposure [27–29]. Our findings indicate that clonal dynamics during CAR-T therapy follow divergent trajectories across distinct mutations in CHIP. *TP53* and *ASXL1* mutations exhibited clonal expansion over time, with *TP53* showing the most pronounced increase compared with all other CHIP alterations. In contrast, *PPM1D* and *DNMT3A* mutations demonstrated progressive reductions in absolute VAF, consistent with negative selection during longitudinal follow-up. Together, these patterns suggest mutation-specific selective pressures acting on DNA damage response genes and epigenetic regulators in the CAR-T context. In particular, inflammatory stress can promote the expansion of CHIP clones, such as *TP53*, increasing the risk of developing myeloid malignancies [30].

Secondary malignancies following CAR T-cell therapy have emerged as an important safety concern following the recent black box warning issued by the United States Food and Drug Administration [31, 32]. Notably, *TP53* mutations in CHIP have been previously linked to inferior survival and clonal expansion of genomically compromised cells, thereby inducing cellular stress mechanisms and activation of inflammatory signaling cascades following chemo-radiation therapy [33]. Inflammation and a tumor-promoting microenvironment may compromise immunotherapy efficacy and predispose patients to immune-related toxicities [26, 34, 35]. Indeed, in our cohort, patients with *TP53*^*mut*^ CHIP were characterized by lower baseline platelet counts and hemoglobin levels, suggesting an interplay between pre-existing cytopenias, post-infusion hematotoxicity, and *TP53*^*mut*^ CHIP. In addition, these patients exhibited a distinct pro-inflammatory baseline state, characterized by elevated CRP and ferritin levels. This systemic inflammatory activation was mirrored by higher baseline CAR-HEMATOTOX scores among *TP53*^*mut*^ CHIP carriers [26, 36]. Our proteomic analysis further confirmed this finding, revealing a pronounced inflammatory profile in patients with *TP53*^*mut*^ CHIP, highlighting a potential biomarker signature that may be relevant for patient monitoring during the first 28 post-CAR-T days. In particular, IL-18, Gal-9, PGF, and MIC-A/B were significantly upregulated in patients characterized by *TP53*^*mut*^ CHIP, hinting toward a marked inflammatory condition and possible immune dysregulation. Elevated IL-18 levels have been shown to potentiate inflammation through the induction of IFNy production and were linked to the development of post-CAR-T cytopenias [26, 37, 38]. This pro-inflammatory environment is further sustained by increased PGF levels, a marker linked to systemic inflammation and poor survival outcomes in a range of pathological diseases, including cancer and particularly in leukemia [39, 40]. Pro-inflammatory conditions are known to drive the transcriptional activation of Galectin-9, which binds to TIM3 and subsequently can promote T-cell exhaustion [41, 42]. Accordingly, we observed significantly elevated PD-L1 levels, confirming the presence of an exhaustion-driven environment when compared to the *TP53*^*wt*^ CHIP cohort [41, 42].

An elevated baseline HT score and reduced CAR T-cell expansion has been consistently associated with adverse clinical outcomes [26, 36]. In line with this, *TP53*^*mut*^ CHIP carriers showed a trend toward reduced survival compared with non-CHIP patients, although the relatively small number of *TP53*^*mut*^ CHIP cases limited the power to demonstrate a statistically robust difference. Importantly, these results were linked to diminished CAR T-cell expansion in patients with *TP53*^mut^ CHIP, which was not seen in patients bearing other CHIP mutations. Taken together, these findings underscore the potential clinical relevance of identifying *TP53*^*mut*^ CHIP at baseline. Accordingly, we aimed to develop a pragmatic risk score capable of discriminating patients with a higher probability of harboring *TP53*^*mut*^ CHIP before treatment. We therefore derived a simple and clinically applicable baseline risk score based on readily available variables, namely age >65 years and CAR-HEMATOTOX score ≥2. A higher baseline score was significantly more frequent among patients with *TP53*^*mut*^ CHIP compared with *TP53*^*wt*^ CHIP patients in our cohort. These findings suggest that this pragmatic scoring system may represent a straightforward tool to select patients for targeted sequencing before CAR-T administration, thereby enabling closer surveillance, particularly with respect to hematologic toxicity and the potential development of MN-pCT.

Importantly, two patients with a pre-existing *TP53*^*mut*^ CHIP subsequently developed a treatment-emergent AML whereas another patient developed a *TP53* mutation at the time of the MDS diagnosis. Together, these findings reinforce the hypothesis that pre-existent *TP53*^*mut*^ CHIP may actively contribute to the pathogenesis of MN-pCT [43]. The occurrence of MN-pCTs ranged from 1.7 to 4.6 years after CAR-T infussion. While this is comparable to conventional therapy, it should be noted that other studies have reported shorter latency after CAR-T compared to ASCT [44]. In the MDS-pCT patient, clonal expansion of *ASXL1* may have altered the bone marrow microenvironment, selecting for *TP53*^*mut*^ clones and promoting secondary malignancy. On the other hand, expansion of pre-existing *TP53* clones suggests a selection process potentially induced by lymphodepletion and CAR-T-mediated inflammation or immune suppression [5, 45]. This process may have created permissive conditions for the outgrowth of previously constrained *NRAS and BCOR* mutations that manifested at the time of AML-pCT diagnosis. In addition, an inflammatory state marked by high levels of IL-18 [38] was associated with an immunosuppressive microenvironment characterized by downregulation of co-stimulatory (TNFRSF9 and TNFRSF4) and anti-tumor immunity molecules (IL-12, TNFSF14) [46–49]. This indicates an inflamed yet immune suppressed setting that may have facilitated the occurrence of secondary malignancies.

This study has several relevant limitations. Its retrospective and single-center nature may limit the generalizability of the findings. Furthermore, the limited number of samples beyond month 6 may introduce immortal time bias, and the small subgroup sizes warrant validation in larger patient populations. A further limitation pertains to cohort heterogeneity and the difficulty of discerning potential confounding of non-CAR-T-related factors. For example, older patients and those who received multiple prior lines of therapy may be more likely to develop cytopenias, exhibit a stronger inflammatory profile, harbor *TP53*^*mut*^ clones, and experience worse survival. For these reasons, we are ultimately unable to discern the causal relationship with the CAR T cells themselves. Finally, targeted NGS was not performed at pre-defined timepoints, resulting in variability in longitudinal clonal assessment.

The outlined findings suggest routine implementation of monitoring for CHIP in CAR-T recipients older than 65 with high-risk baseline CAR-HEMATOTOX scores. When coupled with *TP53*^mut^ CHIP, this high-risk constellation may warrant closer long-term monitoring, as they appear to correlate with unique inflammatory signatures and may be linked to the later development of myeloid neoplasms post-cytotoxic therapy.

In summary, overall CHIP status was not associated with CAR-T-related toxicity or clinical outcomes. In contrast, *TP53*^*mut*^ CHIP was associated with baseline cytopenias and a proinflammatory state, reflected by higher CAR-HEMATOTOX scores and sustained elevation of inflammatory markers during the first 28 days after infusion. Finally, patients who developed MN-pCT exhibited distinct patterns of clonal expansion and inflammatory profiles, highlighting mutation-specific risks in the setting of CAR T-cell therapy.

## Supplementary information

Supplementary materials

Supplementary figure 1

Supplementary figure 2

Supplementary figure 3

Supplementary figure 4

Supplementary figure 5

Supplementary figure 6

Supplementary figure 7

Supplementary figure 8

## Data Availability

The datasets generated for this study are available from the corresponding author upon reasonable request.

## References

[1] Dong Y, Yang T, Zhao M, Song F, Chen R, Zhang M, et al. Safety and efficacy of autologous humanized CD19 CAR-T cell therapy for relapsed/refractory B-cell non-Hodgkin lymphoma. Bone Marrow Transpl. 2025;60:1445–50. 10.1038/s41409-025-02691-2 PMC12583143 40836026

[2] Jacobson CA, Chavez JC, Sehgal AR, William BM, Munoz J, Salles G, et al. Axicabtagene ciloleucel in relapsed or refractory indolent non-Hodgkin lymphoma (ZUMA-5): a single-arm, multicentre, phase 2 trial. Lancet Oncol. 2022;23:91–103. 10.1016/S1470-2045(21)00591-X 34895487

[3] Wang M, Munoz J, Goy A, Locke FL, Jacobson CA, Hill BT, et al. KTE-X19 CAR T-cell therapy in relapsed or refractory Mantle-cell lymphoma. N Engl J Med. 2020;382:1331–42. 10.1056/NEJMoa1914347 PMC7731441 32242358

[4] Rodriguez-Otero P, Ailawadhi S, Arnulf B, Patel K, Cavo M, Nooka AK, et al. Ide-cel or standard regimens in relapsed and refractory multiple myeloma. N Engl J Med. 2023;388:1002–14. 10.1056/NEJMoa2213614 36762851

[5] Rejeski K, Hill JA, Dahiya S, Jain MD. Noncanonical and mortality-defining toxicities of CAR T cell therapy. Nat Med. 2025;31:2132–46. 10.1038/s41591-025-03813-5 40670774

[6] Cordas Dos Santos DM, Tix T, Shouval R, Gafter-Gvili A, Alberge JB, Cliff ERS, et al. A systematic review and meta-analysis of nonrelapse mortality after CAR T cell therapy. Nat Med. 2024;30:2667–78. 10.1038/s41591-024-03084-6 PMC11765209 38977912

[7] Dibas A, Rhiel M, Patel VB, Andrieux G, Boerries M, Cornu TI, et al. Cell-based models of ‘Cytokine Release Syndrome’ endorse CD40L and granulocyte–macrophage colony-stimulating factor knockout in chimeric antigen receptor T cells as mitigation strategy. Cells. 2023;12:2581. 10.3390/cells12212581 PMC10649043 37947658

[8] Khoury JD, Solary E, Abla O, Akkari Y, Alaggio R, Apperley JF, et al. The 5th edition of the World Health Organization Classification of Haematolymphoid Tumours: Myeloid and Histiocytic/Dendritic Neoplasms. Leukemia. 2022;36:1703–19. 10.1038/s41375-022-01613-1 PMC9252913 35732831

[9] Teipel R, Kroschinsky F, Kramer M, Kretschmann T, Egger-Heidrich K, Krüger T, et al. Prevalence and variation of CHIP in patients with aggressive lymphomas undergoing CD19-directed CAR T-cell treatment. Blood Adv. 2022;6:1941–6. 10.1182/bloodadvances.2021005747 PMC8941459 35008107

[10] Gibson CJ, Lindsley RC, Tchekmedyian V, Mar BG, Shi J, Jaiswal S, et al. Clonal hematopoiesis associated with adverse outcomes after autologous stem-cell transplantation for lymphoma. J Clin Oncol. 2017;35:1598–605. 10.1200/JCO.2016.71.6712 PMC5455707 28068180

[11] Avigan ZM, Catlett J, Bodnar S, Pan D, Aleman A, Sheng T, et al. Clonal hematopoiesis and inflammation predict hematologic toxicity and secondary myeloid malignancies after B-cell maturation antigen–directed chimeric antigen receptor T-cell therapy. Clin Cancer Res. 2025;31:4333–44. 10.1158/1078-0432.CCR-25-1587 PMC12521914 40801897

[12] Panagiota V, Kerschbaum JF, Penack O, Stein CM, Arends CM, Koenecke C, et al. Clinical implications and dynamics of clonal hematopoiesis in anti-CD19 CAR T-cell treated patients. HemaSphere. 2023;7:e957. 10.1097/HS9.0000000000000957 PMC10550045 37799345

[13] Gazeau N, Beauvais D, Tilmont R, Srour M, Ferrant E, Safar V, et al. Myeloid neoplasms after CD19-directed CAR T cells therapy in long-term B-cell lymphoma responders, a rising risk over time? Leukemia. 2025;39:1714–22. 10.1038/s41375-025-02605-7 PMC12208870 40275069

[14] Shah MV, Arber DA, Hiwase DK. *TP53*-mutated myeloid neoplasms: 2024 update on diagnosis, risk-stratification, and management. Am J Hematol. 2025;100:88–115. 10.1002/ajh.27655 PMC12067166 40066944

[15] Gustine JN, Branagan AR, Cirstea DD, Rexha F, Han R, Yee AJ, et al. Impact of clonal hematopoiesis on clinical outcomes to BCMA CAR-T in multiple myeloma. Blood Adv. 2025;9:3026–30. 10.1182/bloodadvances.2025015981 PMC12209912 40203191

[16] Miller PG, Sperling AS, Brea EJ, Leick MB, Fell GG, Jan M, et al. Clonal hematopoiesis in patients receiving chimeric antigen receptor T-cell therapy. Blood Adv. 2021;5:2982–6. 10.1182/bloodadvances.2021004554 PMC8361461 34342642

[17] Goldsmith SR, Shouse G, Wong FL, Bosworth A, Iukuridze A, Chen S, et al. Clonal hematopoiesis is associated with severe cytokine release syndrome in patients treated with chimeric antigen receptor T-cell (CART) therapy. Transpl Cell Ther. 2024;30:927.e1–927.e9. 10.1016/j.jtct.2024.06.008 38871057

[18] Carlson SG, Puliafito B, Brown P, Kawai K, Senaratne TN, Lee TD, et al. Impact of clonal hematopoiesis of indeterminate potential (CHIP) on treatment outcomes in multiple myeloma patients undergoing CAR-T cell therapy. Transpl Cell Ther. 2025;31:S236.10.1007/s12185-026-04249-442470580

[19] Saini NY, Swoboda DM, Greenbaum U, Ma J, Patel RD, Devashish K, et al. Clonal hematopoiesis is associated with increased risk of severe neurotoxicity in axicabtagene ciloleucel therapy of large B-cell lymphoma. Blood Cancer Discov. 2022;3:385–93. Sep 6. 10.1158/2643-3230.BCD-21-0177 PMC9445749 35533245

[20] Lee DW, Santomasso BD, Locke FL, Ghobadi A, Turtle CJ, Brudno JN, et al. ASTCT consensus grading for cytokine release syndrome and neurologic toxicity associated with immune effector cells. Biol Blood Marrow Transpl. 2019;25:625–38. 10.1016/j.bbmt.2018.12.758 PMC12180426 30592986

[21] Rejeski K, Wang Y, Hansen DK, Iacoboni G, Bachy E, Bansal R, et al. Applying the EHA/EBMT grading for ICAHT after CAR-T: comparative incidence and association with infections and mortality. Blood Adv. 2024;8:1857–68. 10.1182/bloodadvances.2023011767 PMC11007437 38181508

[22] Liang EC, Rejeski K, Fei T, Albittar A, Huang JJ, Portuguese AJ, et al. Development and validation of an automated computational approach to grade immune effector cell-associated hematotoxicity. Bone Marrow Transpl. 2024;59:910–7. 10.1038/s41409-024-02278-3 PMC11380036 38627450

[23] Rothenberg-Thurley M, Amler S, Goerlich D, Köhnke T, Konstandin NP, Schneider S, et al. Persistence of pre-leukemic clones during first remission and risk of relapse in acute myeloid leukemia. Leukemia. 2018;32:1598–608. 10.1038/s41375-018-0034-z PMC6035153 29472724

[24] Xie Z, Castaneda Puglianini OA, Nishihori T, Grajales-Cruz A, Cheng P, Blue B, et al. Beyond chip: Ccrs predicts prolonged cytopenias and inferior survival following anti-BCMA CAR-T in multiple myeloma. Blood. 2025;146:930–930.

[25] Khelil MB, Arbab A, Srikanthan J, Kovandzic LM, Vergé V, Pagès A, et al. CAR T cell–mediated bone marrow inflammation causes hematotoxicity and favors clonal hematopoiesis. Sci Transl Med. 2025;17:eadu9790. 10.1126/scitranslmed.adu9790 40991725

[26] Rejeski K, Perez A, Iacoboni G, Blumenberg V, Bücklein VL, Völkl S, et al. Severe hematotoxicity after CD19 CAR-T therapy is associated with suppressive immune dysregulation and limited CAR-T expansion. Sci Adv. 2023;9:eadg3919. 10.1126/sciadv.adg3919 PMC10516499 37738350

[27] Jaiswal S, Ebert BL. Clonal hematopoiesis in human aging and disease. Science. 2019;366:eaan4673. 10.1126/science.aan4673 PMC8050831 31672865

[28] Coombs CC, Zehir A, Devlin SM, Kishtagari A, Syed A, Jonsson P, et al. Therapy-related clonal hematopoiesis in patients with non-hematologic cancers is common and associated with adverse clinical outcomes. Cell Stem Cell. 2017;21:374–382.e4. 10.1016/j.stem.2017.07.010 PMC5591073 28803919

[29] Bolton KL, Ptashkin RN, Gao T, Braunstein L, Devlin SM, Kelly D, et al. Cancer therapy shapes the fitness landscape of clonal hematopoiesis. Nat Genet. 2020;52:1219–26. 10.1038/s41588-020-00710-0 PMC7891089 33106634

[30] Chen S, Barajas S, Vemula S, Yang Y, Simpson E, Gao H, et al. Mutant p53 promotes clonal hematopoiesis by generating a chronic inflammatory microenvironment. J Clin Invest. 2026;136:e184285. 10.1172/JCI184285 PMC12867160 41468163

[31] Tix T, Alhomoud M, Shouval R, Cliff ERS, Perales MA, Cordas Dos Santos DM, et al. Second primary malignancies after CAR T-cell therapy: a systematic review and meta-analysis of 5,517 lymphoma and myeloma patients. Clin Cancer Res. 2024;30:4690–700. 10.1158/1078-0432.CCR-24-1798 PMC11546643 39256908

[32] Lorenc R, Shouval R, Flynn JR, Devlin SM, Saldia A, De Abia AL, et al. Subsequent malignancies after CD19-targeted chimeric antigen receptor T cells in patients with lymphoma. Transpl Cell Ther. 2024;30:990–1000. 10.1016/j.jtct.2024.06.027 PMC11427145 38972512

[33] Nead KT, Kim T, Joo L, McDowell TL, Wong JW, Chan ICC, et al. Impact of cancer therapy on clonal hematopoiesis mutations and subsequent clinical outcomes. Blood Adv. 2024;8:5215–24. 10.1182/bloodadvances.2024012929 PMC11530395 38830141

[34] Jain MD, Zhao H, Wang X, Atkins R, Menges M, Reid K, et al. Tumor interferon signaling and suppressive myeloid cells are associated with CAR T-cell failure in large B-cell lymphoma. Blood. 2021;137:2621–33. 10.1182/blood.2020007445 PMC8120145 33512407

[35] Faramand RG, Lee SB, Jain MD, Cao B, Wang X, Rejeski K, et al. Baseline serum inflammatory proteins predict poor CAR T outcomes in diffuse large B-cell lymphoma. Blood Cancer Discov. 2024;5:106–13. 10.1158/2643-3230.BCD-23-0056 PMC10905320 38194367

[36] Rejeski K, Perez A, Sesques P, Hoster E, Berger C, Jentzsch L, et al. CAR-HEMATOTOX: a model for CAR T-cell–related hematologic toxicity in relapsed/refractory large B-cell lymphoma. Blood. 2021;138:2499–513. 10.1182/blood.2020010543 PMC8893508 34166502

[37] Frigault MJ, Graham CE, Berger TR, Ritchey J, Horick NK, El-Jawahri A, et al. Phase 1 study of CAR-37 T cells in patients with relapsed or refractory CD37+ lymphoid malignancies. Blood. 2024;144:1153–67. 10.1182/blood.2024024104 PMC11830985 38781564

[38] Landy E, Carol H, Ring A, Canna S. Biological and clinical roles of IL-18 in inflammatory diseases. Nat Rev Rheumatol. 2024;20:33–47. 10.1038/s41584-023-01053-w PMC12337794 38081945

[39] Ruggiero D, Nutile T, Nappo S, Tirozzi A, Bellenguez C, Leutenegger AL, et al. Genetics of PlGF plasma levels highlights a role of its receptors and supports the link between angiogenesis and immunity. Sci Rep. 2021;11:16821. 10.1038/s41598-021-96256-0 PMC8376970 34413389

[40] Schmidt T, Masouleh BK, Loges S, Cauwenberghs S, Fraisl P, Maes C, et al. Loss or inhibition of stromal-derived PlGF prolongs survival of mice with imatinib-resistant Bcr-Abl1+ leukemia. Cancer Cell. 2011;19:740–53. 10.1016/j.ccr.2011.05.007 21665148

[41] Yang R, Sun L, Li CF, Wang YH, Yao J, Li H, et al. Galectin-9 interacts with PD-1 and TIM-3 to regulate T cell death and is a target for cancer immunotherapy. Nat Commun. 2021;12:832. 10.1038/s41467-021-21099-2 PMC7864927 33547304

[42] Frede J, Poller JC, Shi K, Stuart H, Sotudeh N, Havig C, et al. The endogenous T cell landscape is reshaped by CAR-T cell therapy and predicts treatment response in multiple myeloma. Leukemia. 2025;39:3004–14. 10.1038/s41375-025-02766-5 PMC12634420 40973768

[43] Soerensen JF, Aggerholm A, Kerndrup GB, Hansen MC, Ewald IKL, Bill M, et al. Clonal hematopoiesis predicts development of therapy-related myeloid neoplasms post–autologous stem cell transplantation. Blood Adv. 2020;4:885–92. 10.1182/bloodadvances.2019001157 PMC7065480 32150606

[44] Waldschmidt J, Fandrei D, Hecker JS, Meggendorfer M, Truger M, Reinhard F, et al. Flare of clonal hematopoiesis, TP53 expansion and prior melphalan drive post-CAR-T myeloid disorders in multiple myeloma. Leukemia. 2026;40:1054–7. 10.1038/s41375-026-02941-2 PMC13149010 41876727

[45] Patel SA, Spiegel JY, Dahiya S. Second primary cancer after chimeric antigen receptor–T-cell therapy: a review. JAMA Oncol. 2025;11:174. 10.1001/jamaoncol.2024.5412 39666320

[46] Singh R, Kim YH, Lee SJ, Eom HS, Choi BK. 4-1BB immunotherapy: advances and hurdles. Exp Mol Med. 2024;56:32–9. 10.1038/s12276-023-01136-4 PMC10834507 38172595

[47] Redmond WL, Ruby CE, Weinberg AD. The role of OX40-mediated co-stimulation in T-cell activation and survival. Crit Rev Immunol. 2009;29:187–201. 10.1615/critrevimmunol.v29.i3.10 PMC3180959 19538134

[48] Jung K, Ha JH, Kim JE, Kim JA, Kim YJ, Kim CH, et al. Heterodimeric Fc-fused IL12 shows potent antitumor activity by generating memory CD8^+^ T cells. OncoImmunology. 2018;7:e1438800. 10.1080/2162402X.2018.1438800 PMC5993495 29900039

[49] Zhang N, Liu X, Qin J, Sun Y, Xiong H, Lin B, et al. LIGHT/TNFSF14 promotes CAR-T cell trafficking and cytotoxicity through reversing immunosuppressive tumor microenvironment. Mol Ther. 2023;31:2575–90. 10.1016/j.ymthe.2023.06.015 PMC10491984 37408308

