# Supplementary material for: TP53-mutant CHIP defines a pro-inflammatory phenotype and predicts adverse outcomes after CAR T-cell therapy
Source: Leukemia. 2026 Aug 24;40(11):2540–51. doi: 10.1038/s41375-026-03083-1 (PMC13713554; doi:10.1038/s41375-026-03083-1)
Supplement: Supplementary file 1 [file 41375_2026_3083_MOESM1_ESM.pdf]

SUPPLEMENTARY MATERIALS

SUPPLEMENTARY METHODS:

Serum proteomic analysis

Briefly, pairs of oligonucleotide-conjugated antibodies bind to each target protein allowing the process to hybridize and extend to form a unique DNA sequence that identifies the protein. To test protein-level differences by patient group (CHIP vs no CHIP) and time point, per-protein linear regression models were fitted including CHIP status and sampling time point as fixed effects to assess differences in protein abundance between groups, using Olink Insight Stat Analyzer. Plots were generated using the ggplot2 and heatmap packages (RStudio v4.5.1). Protein concentrations were expressed in NPX units on a log2 scale, where a one-unit change denotes a doubling of abundance. For proteomic analyses, per-protein statistical testing was performed using Olink Insight – Stat Analyzer, with p-values adjusted for multiple hypothesis testing using the Benjamini-Hochberg method.

SUPPLEMENTARY TABLES:

Supplementary table 1: Summary of published data on CAR T-cells and CHIP

Study	Study population	CHIP Prevalence	CRS (*≥2)		ICANS (any grade)		Prolonged Cytopenias		PFS / EFS		OS		MN-pCT reported	Findings
			CHIP	No CHIP	CHIP	No CHIP	CHIP	No CHIP	CHIP	No CHIP	CHIP	No CHIP		
Teipel et al., Blood Advances (2022)	32 r/r B-cell NHL	34%	no difference**		no difference**		no difference**		median EFS not reached	median EFS 77 days	median OS not reached	median OS 265 days	no	• No correlation between CHIP and CRS/ICANS, cytopenia or inflammation<br>• Better event free survival and overall survival after CAR T-cells in CHIP patients
Panagiota et al., HemaSphere (2022)	100 r/r B-cell NHL / 5 ALL	56.40%	67.7%	72.9%	33.9%	27.1%	67.4%	60.7%	1-year PFS 35%	1-year PFS 46%	1-years OS 53%	1-years OS 55%	no	• No association between CHIP and toxicities or prolonged cytopenias<br>• No influence of CHIP on CAR T cell response<br>• No impact of CHIP on long-term outcomes
Gustine et al., Blood Advances (2025)	104 MM	55%	82%	83%	18%	30%	Anaemia n=14 / Thrombocytopenia n= 13 / Neutropenia n=15	Anaemia n=0 / Thrombocytopenia n= 0 / Neutropenia n=0	12.0	11.8	24.6	38.1	no	• CHIP did not affect response, survival, or CRS/ICANS<br>• CHIP was associated with prolonged cytopenias and delayed hematopoietic recovery
Miller et al., Blood Advances (2021)	154 NHL and MM	48%	77.8%*	45.9%*	NA	NA	NA	NA	median PFS 25.6 mo	median PFS 21.5 mo	no difference**		yes	• Higher complete response in CHIP patients<br>• Increased severe CRS in CHIP patients <60<br>• No association of CHIP with survival
Goldsmith et al., Transplantation and Cellular Therapy (2024)	38 LBCL / 2 MM / 2 MCL / 20 tNHL / 1 FL	24%	60%*	28%*	no difference**		no difference**		no difference**		no difference**		yes	• CHIP associated with grade ≥2 CRS<br>• No association between CHIP and ICANS, cytopenias or survival
Carlson et al., Transplantation and Cellular Therapy (2025 / *meeting abstract*)	51 MM	33%	100%	65%	41%	21%	no difference**		no difference**		no difference**		no	• CHIP associated with severe CRS<br>• No association between CHIP and response rate, survival or ICANS
Saini et al., Blood Cancer Discovery (2022)	114 LBCL	33%	47.6%*	45.8%*	52.3%*	41.7%*	no difference**		no difference**		no difference**		yes	• CHIP associated with severe ICANS and CRS<br>• No association between CHIP and response rate or survival
Waldschmidt et al., Leukemia (2026)	179 r/r MM	NA	NA	NA	NA	NA	NA	NA	NA	NA	NA	NA	yes	• Origin of MN-pCAR can be traced back to melphalan treatment<br>• MN-pCT arose from pre-existing CH clones with a fast pos CAR T expansion
Rappa and Ziemann et al., Leukemia (2026)	70 LBCL / 19 MM / 13 MCL / 2 FL	37.50%	53.80%	38.50%	35.90%	38.50%	Grade ≥3 ICAHT = 9%	Grade ≥3 ICAHT = 16%	median PFS 8.2 mo	median PFS 5.0 mo	median OS 18.1 mo	median OS 21.9 mo	yes	• CHIP per se does not impact toxicity, inflammation or survival after CAR-T<br>• *TP53*^{mut} CHIP defines a clinically distinct high-risk subgroup with baseline cytopenias, a unique pro-inflammatory serum signature, and markedly impaired CAR T expansion<br>• First clinical score validated to prospectively stratify patients at risk of harboring *TP53*-mutant CHIP prior to CAR T<br>• MN-pCAR clonal dynamics post CAR T characterized with paired longitudinal profiling

* significant
** % not provided

Supplementary table 2: Genes included in the targeted sequencing panel used for CHIP identification

ANKRD26	exon 1	*GATA3*	*exons 4-6*	*PRPF8*	*entire CDS*
ASXL1	exon 13	*GNB1*	*exons 3-11*	*PTPN11*	*exons 3,13*
ASXL2	exon 12,13	*HNRNPK*	*entire CDS*	*PTPRT*	*entire CDS*
BCOR	entire CDS	*HRAS*	*exons 2,3*	*RAD21*	*entire CDS*
BCORL1	entire CDS	*IDH1*	*exon 4,7*	*RUNX1*	*exons 4-9*
BRAF	exons 11,12,15	*IDH2*	*exon 4,7*	*SETBP1*	*exon 4*
BRCC3	entire CDS	*IL7R*	*exon 6*	*SF3B1*	*exons 14-16*
CALR	exon 9	*IL7R*	*exon 6*	*SH3B3*	*exons 2-8*
CBL	exons 7,8,9	*JAK1*	*exons 13-15*	*SRP72*	*entire CDS*
CCND1	exon5	*JAK2*	*exons 10-25*	*SRSF2*	*exon 1*
CCND2	exon5	*JAK3*	*exons 3-5, 10-16*	*STAG2*	*entire CDS*
CDKN2A	entire CDS	*KIT*	*exons 2,8,9,10-12,17*	*STAT3*	*exons 19-21*
CEBPA	exon 1	*KRAS*	*exons 2-4*	*STAT5B*	*exon 16*
CSF3R	exons 14-17	*MPL*	*exons 1-12*	*TERC*	*entire CDS*
CSFR1	exons 7, 22	*MYC*	*exon 2*	*TERT*	*exons 1-16*

CSNK1A1	exons 4,3	*MYD88*	*exons 3-5*	*TET2*	*entire CDS*
CXCR4	exon 1	*NF1*	*entire CDS*	*TP53*	*entire CDS*
DDX41	entire CDS	*NFE2*	*exons 2,3*	*U2AF1*	*exons 2,6*
DNMT3A	exons 7-23	*NOTCH1*	*exons 26-28,34*	*U2AF2*	*entire CDS*
ETNK1	exon 3	*NPM1*	*exons 10,11*	*WT1*	*entire CDS*
ETV6	entire CDS	*NRAS*	*exons 2-4*	*XPO1*	*exon 16*
EZH2	entire CDS	*NT5C2*	*entire CDS*	*ZBTB33*	*exon 2*
FLT3	exons 13-16, 20	*PHF6*	*entire CDS*	*ZBTB7A*	*exons 2,3*
GATA1	exons 2,3	*PIGA*	*exons 1-6*	*ZRSR2*	*entire CDS*
GATA2	exons 2-6	*PPM1D*	*exon 6*		

Supplementary table 3: J-T trend test depicting the trend of WBCs in patients who experience CHIP expansion or no expansion

**Jonckheere-Terpstra Trend Test**						
Monotonic trend in cell counts across ordered timepoint, stratified by CHIP Expansion						
Parameter	CHIP Expansion	N	n per timepoint	JT statistic	p-value	H1 direction
Decline						
ANC [G/l]	No	133	61 / 30 / 42	1117	**< 0.001**	decreasing
ANC [G/l]	Yes	130	60 / 31 / 39	1090.5	**< 0.001**	decreasing
Hemoglobin [g/dl]	No	165	67 / 42 / 56	4067.5	0.11	decreasing
Hemoglobin [g/dl]	Yes	144	61 / 36 / 47	2366.5	**< 0.001**	decreasing
Leucocyte count [G/l]	No	165	67 / 42 / 56	2223	**< 0.001**	decreasing
Leucocyte count [G/l]	Yes	144	61 / 36 / 47	1628.5	**< 0.001**	decreasing
Platelet count [G/l]	No	151	64 / 41 / 46	2708	**< 0.001**	decreasing
Platelet count [G/l]	Yes	140	61 / 34 / 45	1805	**< 0.001**	decreasing
Recovery (d7 to d30)						
ANC [G/l]	No	191	95 / 47 / 49	7184.5	**< 0.001**	increasing
ANC [G/l]	Yes	172	91 / 50 / 31	4756	0.19	increasing
Hemoglobin [g/dl]	No	209	105 / 54 / 50	6149.5	0.92	increasing
Hemoglobin [g/dl]	Yes	180	91 / 53 / 36	5335.5	0.19	increasing
Leucocyte count [G/l]	No	209	105 / 54 / 50	8188	**0.001**	increasing
Leucocyte count [G/l]	Yes	180	91 / 53 / 36	4714.5	0.79	increasing
Platelet count [G/l]	No	147	82 / 40 / 25	2061.5	1	increasing
Platelet count [G/l]	Yes	146	80 / 40 / 26	2345	1	increasing
JT = Jonckheere-Terpstra statistic (permutation, nperm=2000, set.seed(42)). N per timepoint in bin order						

CHIP, clonal hematopoiesis of indeterminate potential; ANC, absolute neutrophil count

Supplementary table 4: Demographics, clinical characteristics and laboratory parameters of *TP53*^{mut} CHIP versus *TP53*^{wt} CHIP patients.

Variable	All CHIP patients (N=39)[1]	*TP53*^{mut} CHIP (n=8)[1]	*TP53*^{wt} CHIP (n=31)[1]	p value
**Clinical characteristics**				
**Age, years**	66 [62; 73]	69 [63; 73]	66 [62; 74]	0.499[2]
**Sex**				0.432[3]
female	18 (46%)	5 (63%)	13 (42%)	
male	21 (54%)	3 (38%)	18 (58%)	
**Diagnosis**				0.086[3]
B-NHL	29 (74%)	8 (100%)	21 (68%)	
MM	10 (26%)	0 (0%)	10 (32%)	
**CAR-T product**				0.235[3]
Axi-cel	16 (41%)	3 (38%)	13 (42%)	
Liso-cel	4 (10%)	1 (13%)	3 (9.7%)	
Cilta-cel	2 (5.1%)	0 (0%)	2 (6.5%)	
Tisa-cel	6 (15%)	3 (38%)	3 (9.7%)	
Brexu-cel	3 (7.7%)	1 (13%)	2 (6.5%)	
Ide-cel	8 (21%)	0 (0%)	8 (26%)	
**Prior lines of therapy**	3 [2; 5]	4 [2; 4]	3 [2; 5]	0.481[2]
**Prior auto-HCT**	16 (41%)	3 (38%)	13 (42%)	p > 0.999[3]
**Prior exposure to alkylating agents**	37 (95%)	8 (100%)	29 (94%)	p > 0.999[3]
**Time from initial diagnosis to CAR T, months**	48.3 [15.6; 76.5]	49.1 [22.2; 60.2]	36.9 [15.6; 94.4]	0.772[2]
**Hematotox score**	1 [0; 3]	3 [3; 6]	1 [0; 2]	**0.003[2]**
**Laboratory parameters at lymphodepletion**				
**ANC x 10^9/L**	2.45 [1.62; 3.57]	1.98 [1.29; 2.38]	2.75 [1.67; 3.96]	0.074[2]
**Platelets x 10^9/L**	176 [90; 276]	91 [38; 152]	207 [106; 280]	**0.016[2]**
**Hemoglobin g/dL**	10.00 [8.70; 11.30]	8.85 [7.50; 9.85]	10.30 [9.00; 11.50]	**0.012[2]**
**CRP mg/L**	0.80 [0.20; 2.70]	2.30 [1.35; 4.75]	0.30 [0.20; 2.30]	**0.015[2]**
**Ferritin mg/L**	374 [107; 1,062]	1,256 [703; 3,023]	236 [93; 518]	**0.001[2]**
**LDH U/L**	241 [188; 383]	349 [203; 502]	231 [184; 340]	0.361[2]

[1]Median [Q1; Q3]; n (%)

[2]Wilcoxon rank-sum test

[3]Fisher's exact test

CHIP, clonal hematopoiesis of indeterminate potential; mut, mutant; wt, wild type; B-NHL, B-cell Non-Hodgkin lymphoma; MM, multiple myeloma; Axi-cel, Axicabtagene ciloleucel; Liso-cel, Lisocabtagene maraleucel; Cilta-cel, Ciltacabtagene autoleucel; Tisa-cel, Tisagenlecleucel, Brexu-cel, Brexucabtagene autoleucel; Ide-cel, Idecabtagene vicleucel; Auto-HCT, autologous hematopoietic cell transplantation; ANC, absolute neutrophil count; CRP, C-reactive protein; LDH, lactate dehydrogenase.

Supplementary table 5: Age and HT derived risk score's measures.

Predictor	AUC	AUC 95CI	Sensitivity	Specificity	p value
**Age >65**	0.68	0.51-0.85	0.75	0.61458	**0.023**
**HT score ≥2**	0.72	0.59-0.85	0.875	0.5625	**0.009**
**Age >65 + HT score ≥2**	0.8	0.67-0.92	0.625	0.84375	**0.001**

HT, Hematotox; AUC, area under the curve

Supplementary table 6: Contingency table of the study population included for the simple risk score calculation.

	***TP53*wt**	***TP53*mut**	**Tot**
**Risk score =2**	15	5	20
**Risk score ≤1**	81	3	84
**Tot**	96	8	

mut, mutant; wt, wild type

SUPPLEMENTARY FIGURES:

Supplementary figure 1: A), C), E) PFS and B), D), F) OS of patients diagnosed with LBCL (n=70), MM (n=19) and MCL (n=13), respectively. Patients characterized by CHIP mutations are depicted in red and patients without CHIP mutations in blue. PFS and OS were compared using the Kaplan-Meier estimates and the log-rank test; hazard ratios were estimated using Cox proportional hazards model.

Supplementary figure 2: A), C), E), G), I), PFS and B), D), F), H), L) OS of patients treated with Axi-cel (n=46), Tisa-cel (n=16), Ide-cel (n=15), Brexu-cel (n=13), and Liso-cel (n=10), respectively. Patients characterized by CHIP mutations are depicted in red and patients without CHIP mutations in blue. PFS and OS were compared using the Kaplan-Meier

estimates and the log-rank test; hazard ratios were estimated using Cox proportional hazards model.

Supplementary figure 3: Volcano plot showing the estimated mean difference between patient with or without CHIP (x-axis) and corresponding -log10 (y-axis, adjusted p value = 3) between day 0 and day 28.

Supplementary figure 4: A) CAR expansion trajectory stratified by CHIP status. B) AUC of CAR T-cell-expansion by CHIP status. In red CHIP and in blue no CHIP cohorts.

Supplementary figure 5: VAF change in individuals bearing at least two mutations. The y-axis depicts the change in VAF percentage while the x-axis shows the follow up time in months.

Supplementary figure 6: A) Differential protein expression of the 19 proteins differing between *TP53*^{mut} CHIP patients (left) and *TP53*^{wt} CHIP patients (right). The x-axis represents the estimated NPX difference between the two groups, with color indicating the corresponding -log10 p values. B) Timepoint analysis showing the inflammatory signature of *TP53*^{wt} CHIP patients at day 0, 2 to 7, 14 and 28. Heatmap color scale indicates relative protein concentration; red denotes higher and blue denotes lower relative expression. C) PFS and D) OS of patients with *TP53*^{mut} CHIP (n=8) or *TP53*^{wt} CHIP (n=31). PFS and OS were compared using the Kaplan-Meier estimates and the log-rank test; hazard ratios were estimated using Cox proportional hazards model.

Supplementary figure 7: Treatment history of CAR T patients diagnosed with MN-pCT. Day 0 refers to the time of CAR T cell indication.

Supplementary figure 8: A) Toxicity grades for CRS, ICANS, early and late ICAHT in patients characterized by MN-pCT. Toxicities with grade ≥3 were considered severe. B), C) and D) Longitudinal plots describing ferritin (in blue) and CRP (in red) in MN-pCT patients (MDS-pCT #1, AML-pCT #2 and #3 respectively). The x-axis represents the days relative to CAR T-cell infusion. The left y-axis represents the ferritin count while the right y-axis represents the CRP count.
