# Supplementary material for: TP53-mutant CHIP defines a pro-inflammatory phenotype and predicts adverse outcomes after CAR T-cell therapy
Source: Leukemia. 2026 Aug 24;40(11):2540–51. doi: 10.1038/s41375-026-03083-1 (PMC13713554; doi:10.1038/s41375-026-03083-1)
Supplement: Supplementary file 2 [file 41375_2026_3083_MOESM2_ESM.pdf]

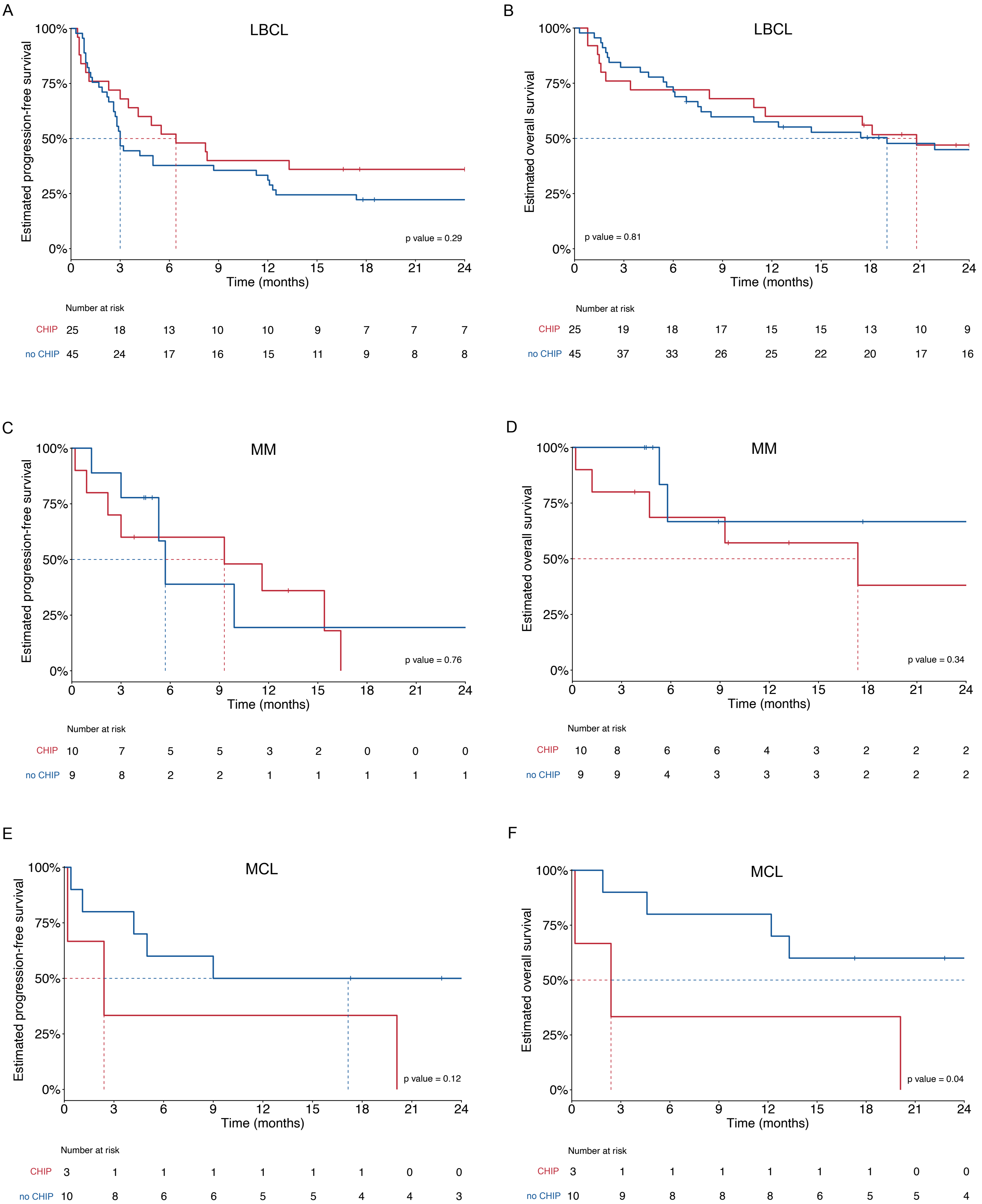
A
LBCL
Estimated progression-free survival
100%
75%
50%
25%
0%
0 3 6 9 12 15 18 21 24
Time (months)
p value = 0.29
Number at risk
CHIP 25 18 13 10 10 9 7 7 7
no CHIP 45 24 17 16 15 11 9 8 8
B
LBCL
Estimated overall survival
p value = 0.81
Number at risk
CHIP 25 19 18 17 15 15 13 10 9
no CHIP 45 37 33 26 25 22 20 17 16
C
MM
Estimated progression-free survival
p value = 0.76
Number at risk
CHIP 10 7 5 5 3 2 0 0 0
no CHIP 9 8 2 2 1 1 1 1 1
D
MM
Estimated overall survival
p value = 0.34
Number at risk
CHIP 10 8 6 6 4 3 2 2 2
no CHIP 9 9 4 3 3 3 2 2 2
E
MCL
Estimated progression-free survival
p value = 0.12
Number at risk
CHIP 3 1 1 1 1 1 1 0 0
no CHIP 10 8 6 6 5 5 4 4 3
F
MCL
Estimated overall survival
p value = 0.04
Number at risk
CHIP 3 1 1 1 1 1 1 0 0
no CHIP 10 9 8 8 8 6 5 5 4
