# Supplementary material for: TP53-mutant CHIP defines a pro-inflammatory phenotype and predicts adverse outcomes after CAR T-cell therapy
Source: Leukemia. 2026 Aug 24;40(11):2540–51. doi: 10.1038/s41375-026-03083-1 (PMC13713554; doi:10.1038/s41375-026-03083-1)
Supplement: Supplementary file 3 [file 41375_2026_3083_MOESM3_ESM.pdf]

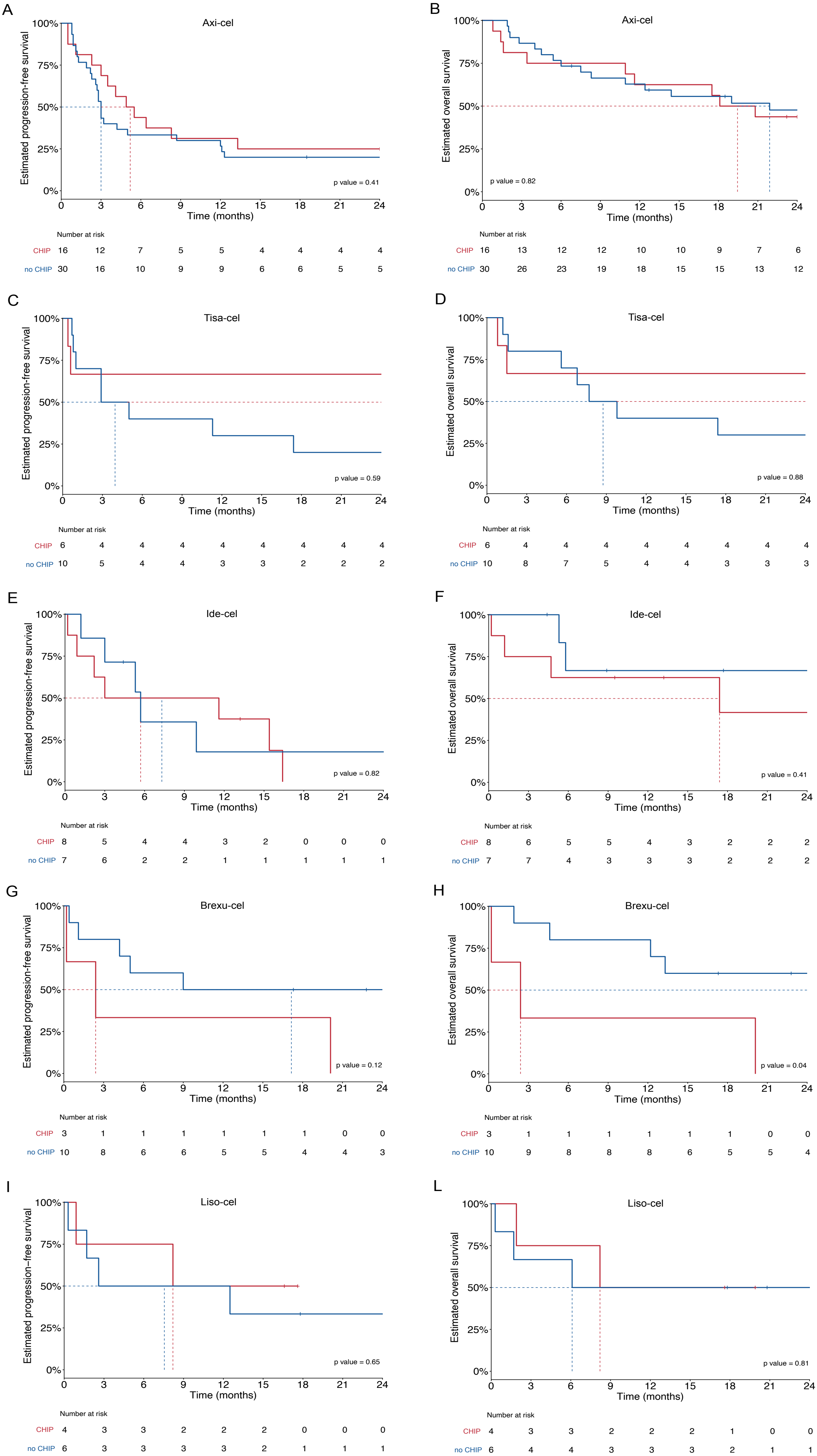
A
Axi-cel
Estimated progression-free survival
100%
75%
50%
25%
0%
p value = 0.41
0 3 6 9 12 15 18 21 24
Time (months)
Number at risk
CHIP 16 12 7 5 5 4 4 4 4
no CHIP 30 16 10 9 9 6 6 5 5
B
Axi-cel
Estimated overall survival
100%
75%
50%
25%
0%
p value = 0.82
0 3 6 9 12 15 18 21 24
Time (months)
Number at risk
CHIP 16 13 12 12 10 10 9 7 6
no CHIP 30 26 23 19 18 15 15 13 12
C
Tisa-cel
Estimated progression-free survival
100%
75%
50%
25%
0%
p value = 0.59
0 3 6 9 12 15 18 21 24
Time (months)
Number at risk
CHIP 6 4 4 4 4 4 4 4 4
no CHIP 10 5 4 4 3 3 2 2 2
D
Tisa-cel
Estimated overall survival
100%
75%
50%
25%
0%
p value = 0.88
0 3 6 9 12 15 18 21 24
Time (months)
Number at risk
CHIP 6 4 4 4 4 4 4 4 4
no CHIP 10 8 7 5 4 4 3 3 3
E
Ide-cel
Estimated progression-free survival
100%
75%
50%
25%
0%
p value = 0.82
0 3 6 9 12 15 18 21 24
Time (months)
Number at risk
CHIP 8 5 4 4 3 2 0 0 0
no CHIP 7 6 2 2 1 1 1 1 1
F
Ide-cel
Estimated overall survival
100%
75%
50%
25%
0%
p value = 0.41
0 3 6 9 12 15 18 21 24
Time (months)
Number at risk
CHIP 8 6 5 5 4 3 2 2 2
no CHIP 7 7 4 3 3 3 2 2 2
G
Brexu-cel
Estimated progression-free survival
100%
75%
50%
25%
0%
p value = 0.12
0 3 6 9 12 15 18 21 24
Time (months)
Number at risk
CHIP 3 1 1 1 1 1 1 0 0
no CHIP 10 8 6 6 5 5 4 4 3
H
Brexu-cel
Estimated overall survival
100%
75%
50%
25%
0%
p value = 0.04
0 3 6 9 12 15 18 21 24
Time (months)
Number at risk
CHIP 3 1 1 1 1 1 1 0 0
no CHIP 10 9 8 8 8 6 5 5 4
I
Liso-cel
Estimated progression-free survival
100%
75%
50%
25%
0%
p value = 0.65
0 3 6 9 12 15 18 21 24
Time (months)
Number at risk
CHIP 4 3 3 2 2 2 0 0 0
no CHIP 6 3 3 3 3 2 1 1 1
L
Liso-cel
Estimated overall survival
100%
75%
50%
25%
0%
p value = 0.81
0 3 6 9 12 15 18 21 24
Time (months)
Number at risk
CHIP 4 3 3 2 2 2 1 0 0
no CHIP 6 4 4 3 3 3 2 1 1
