# Supplementary material for: TP53-mutant CHIP defines a pro-inflammatory phenotype and predicts adverse outcomes after CAR T-cell therapy
Source: Leukemia. 2026 Aug 24;40(11):2540–51. doi: 10.1038/s41375-026-03083-1 (PMC13713554; doi:10.1038/s41375-026-03083-1)
Supplement: Supplementary file 4 [file 41375_2026_3083_MOESM4_ESM.pdf]

A

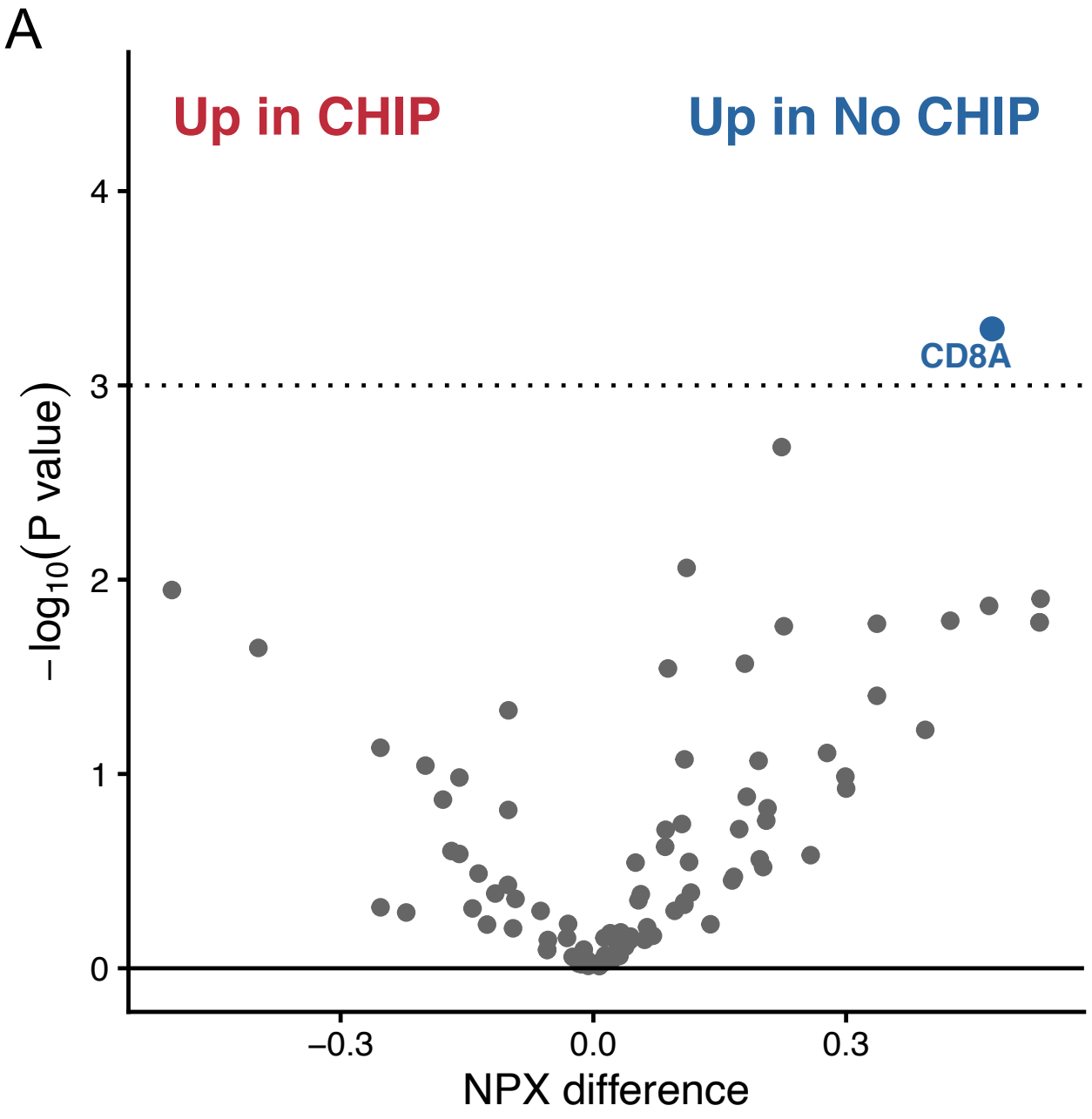

Up in CHIP
Up in No CHIP
CD8A
4
3
2
1
0
−log10(P value)
−0.3
0.0
0.3
NPX difference
