# Supplementary material for: TP53-mutant CHIP defines a pro-inflammatory phenotype and predicts adverse outcomes after CAR T-cell therapy
Source: Leukemia. 2026 Aug 24;40(11):2540–51. doi: 10.1038/s41375-026-03083-1 (PMC13713554; doi:10.1038/s41375-026-03083-1)
Supplement: Supplementary file 5 [file 41375_2026_3083_MOESM5_ESM.pdf]

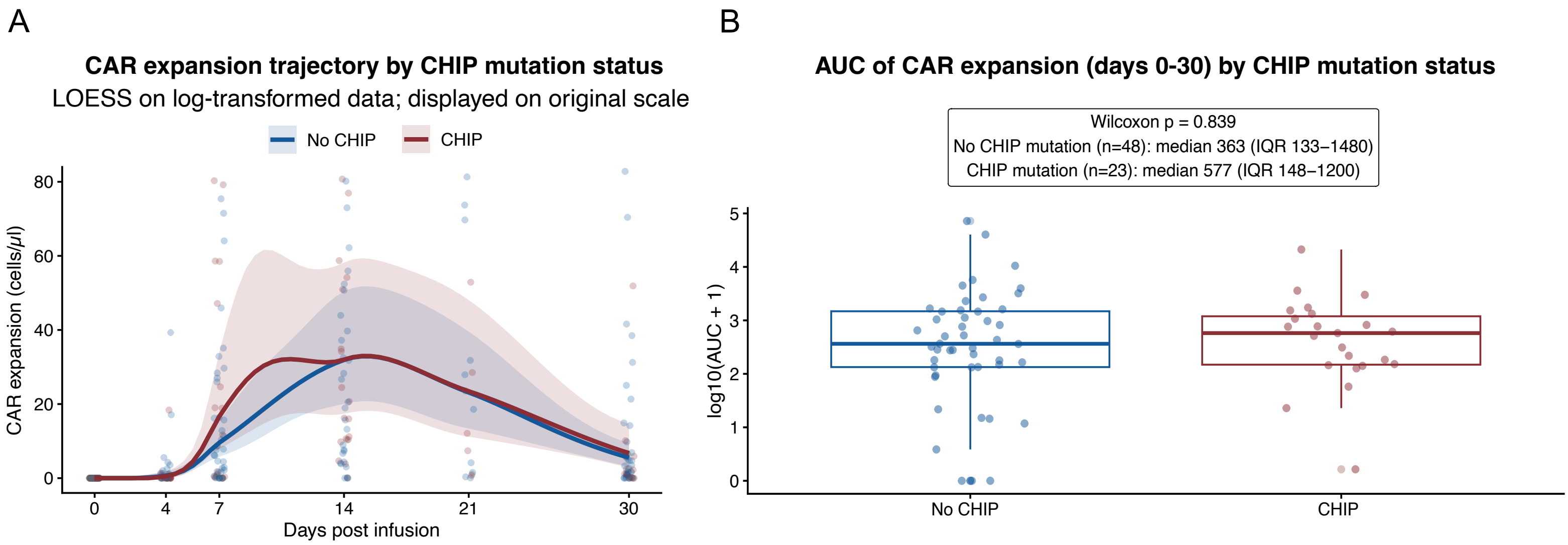
A
CAR expansion trajectory by CHIP mutation status
LOESS on log-transformed data; displayed on original scale
No CHIP
CHIP
CAR expansion (cells/μl)
80
60
40
20
0
0
4
7
14
21
30
Days post infusion
B
AUC of CAR expansion (days 0-30) by CHIP mutation status
Wilcoxon p = 0.839
No CHIP mutation (n=48): median 363 (IQR 133–1480)
CHIP mutation (n=23): median 577 (IQR 148–1200)
log10(AUC + 1)
5
4
3
2
1
0
No CHIP
CHIP
