# Supplementary material for: TP53-mutant CHIP defines a pro-inflammatory phenotype and predicts adverse outcomes after CAR T-cell therapy
Source: Leukemia. 2026 Aug 24;40(11):2540–51. doi: 10.1038/s41375-026-03083-1 (PMC13713554; doi:10.1038/s41375-026-03083-1)
Supplement: Supplementary file 6 [file 41375_2026_3083_MOESM6_ESM.pdf]

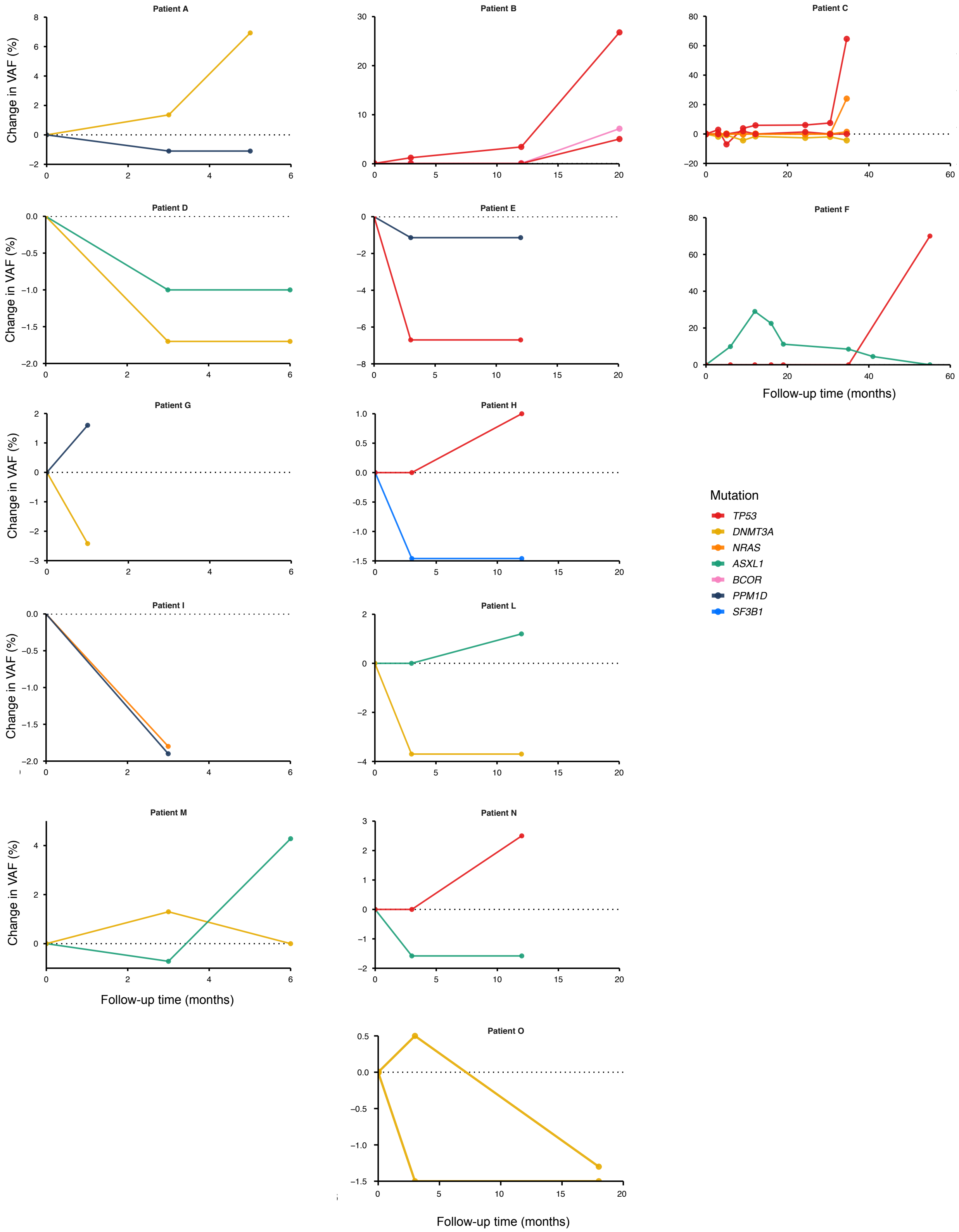
Patient A
Patient B
Patient C
Patient D
Patient E
Patient F
Patient G
Patient H
Patient I
Patient L
Patient M
Patient N
Patient O
Change in VAF (%)
Follow-up time (months)
Mutation
TP53
DNMT3A
NRAS
ASXL1
BCOR
PPM1D
SF3B1
