# Supplementary material for: TP53-mutant CHIP defines a pro-inflammatory phenotype and predicts adverse outcomes after CAR T-cell therapy
Source: Leukemia. 2026 Aug 24;40(11):2540–51. doi: 10.1038/s41375-026-03083-1 (PMC13713554; doi:10.1038/s41375-026-03083-1)
Supplement: Supplementary file 7 [file 41375_2026_3083_MOESM7_ESM.pdf]

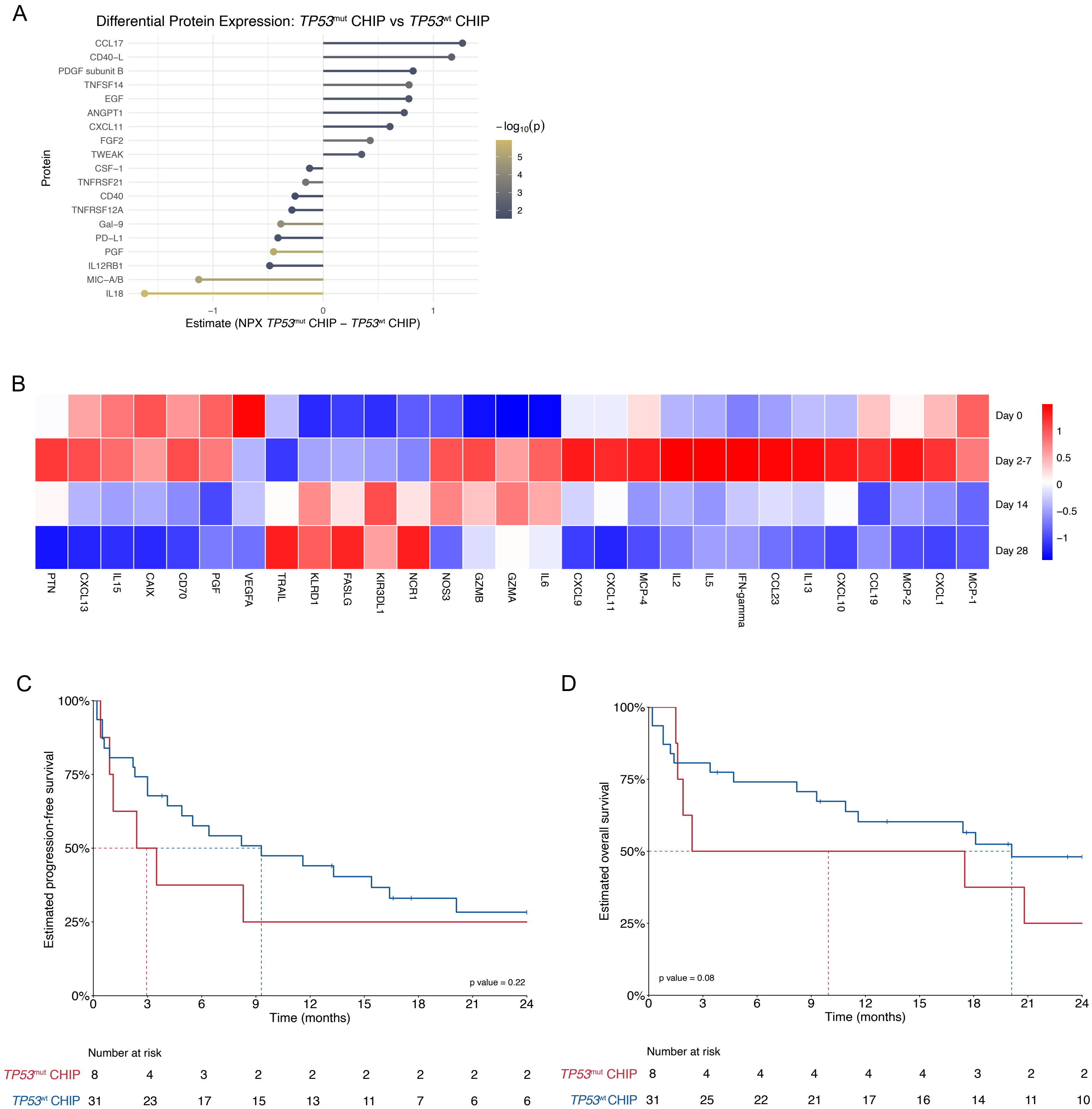
A
Differential Protein Expression: TP53mut CHIP vs TP53wt CHIP
Protein
CCL17
CD40−L
PDGF subunit B
TNFSF14
EGF
ANGPT1
CXCL11
FGF2
TWEAK
CSF−1
TNFRSF21
CD40
TNFRSF12A
Gal−9
PD−L1
PGF
IL12RB1
MIC−A/B
IL18
−1
0
1
Estimate (NPX TP53mut CHIP − TP53wt CHIP)
−log10(p)
5
4
3
2
B
Day 0
Day 2-7
Day 14
Day 28
1
0.5
0
−0.5
−1
PTN
CXCL13
IL15
CAIX
CD70
PGF
VEGFA
TRAIL
KLRD1
FASLG
KIR3DL1
NCR1
NOS3
GZMB
GZMA
IL6
CXCL9
CXCL11
MCP-4
IL2
IL5
IFN-gamma
CCL23
IL13
CXCL10
CCL19
MCP-2
CXCL1
MCP-1
C
Estimated progression-free survival
100%
75%
50%
25%
0%
0
3
6
9
12
15
18
21
24
Time (months)
p value = 0.22
D
Estimated overall survival
100%
75%
50%
25%
0%
0
3
6
9
12
15
18
21
24
Time (months)
p value = 0.08
Number at risk
TP53mut CHIP 8 4 3 2 2 2 2 2 2
TP53wt CHIP 31 23 17 15 13 11 7 6 6
Number at risk
TP53mut CHIP 8 4 4 4 4 4 3 2 2
TP53wt CHIP 31 25 22 21 17 16 14 11 10
