# Supplementary material for: TP53-mutant CHIP defines a pro-inflammatory phenotype and predicts adverse outcomes after CAR T-cell therapy
Source: Leukemia. 2026 Aug 24;40(11):2540–51. doi: 10.1038/s41375-026-03083-1 (PMC13713554; doi:10.1038/s41375-026-03083-1)

Treatment history of *TP53* mutated myeloid diseases

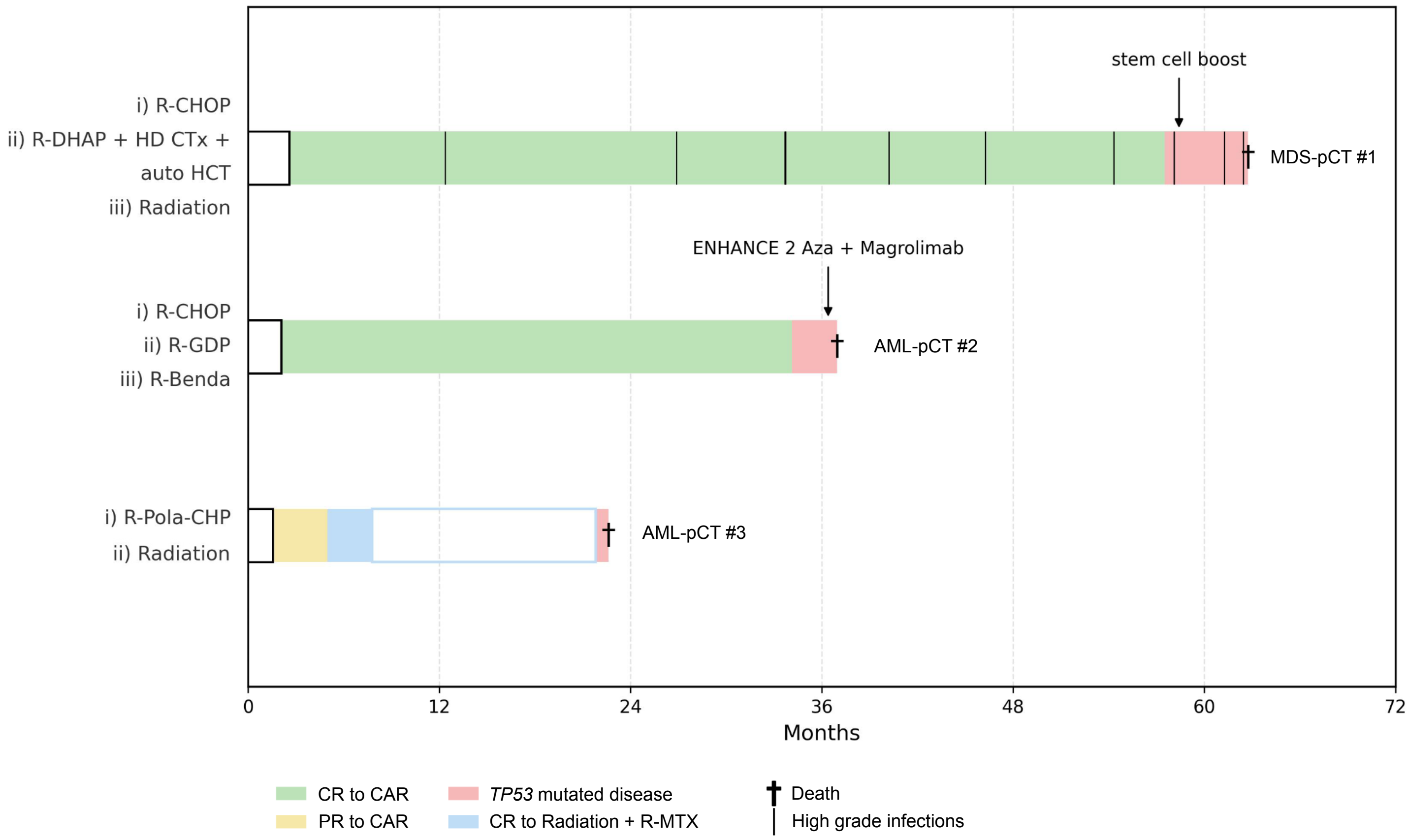

Supplement: Supplementary file 8 [file 41375_2026_3083_MOESM8_ESM.pdf]
