# Supplementary material for: TP53-mutant CHIP defines a pro-inflammatory phenotype and predicts adverse outcomes after CAR T-cell therapy
Source: Leukemia. 2026 Aug 24;40(11):2540–51. doi: 10.1038/s41375-026-03083-1 (PMC13713554; doi:10.1038/s41375-026-03083-1)
Supplement: Supplementary file 9 [file 41375_2026_3083_MOESM9_ESM.pdf]

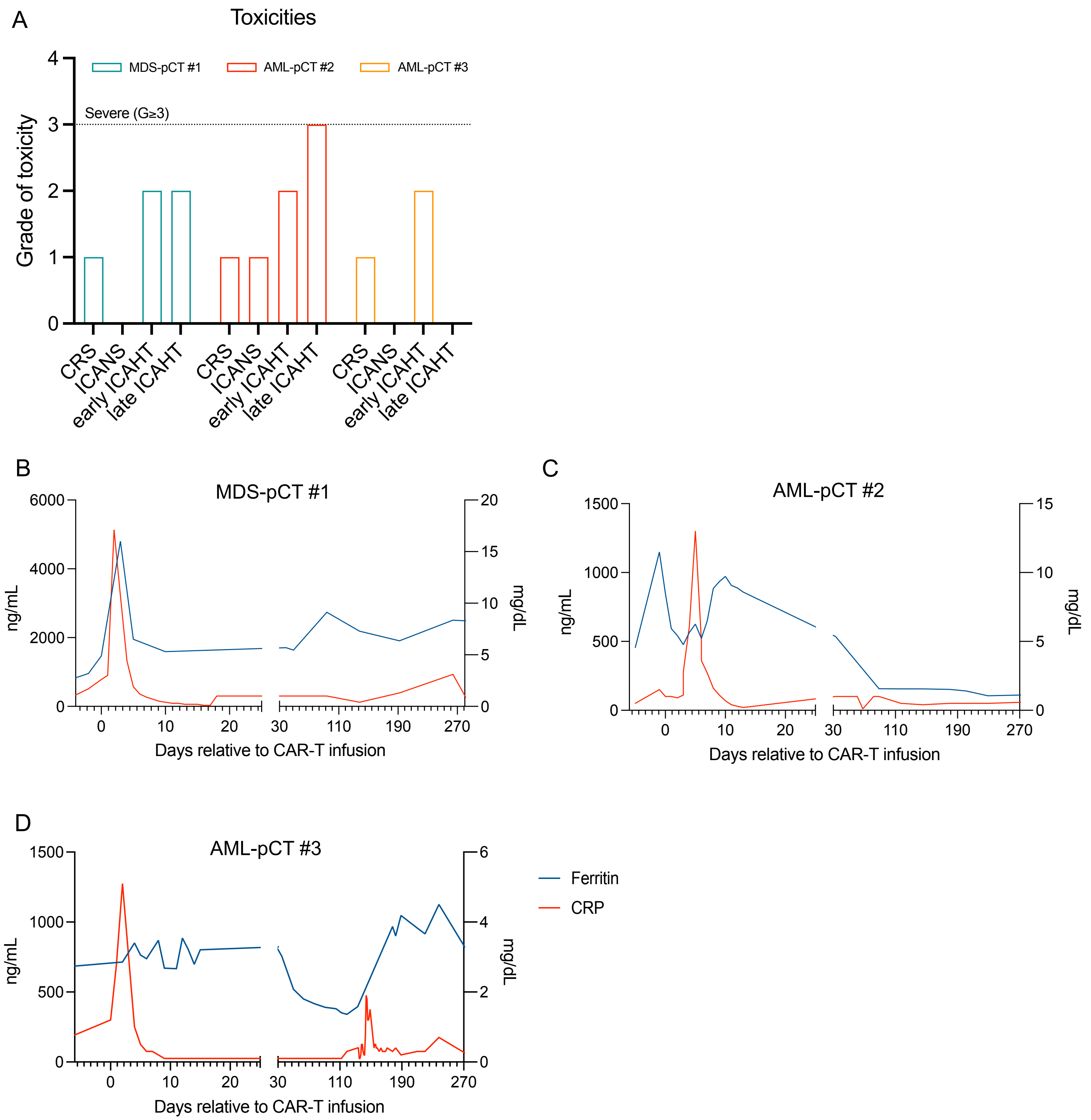
A
Toxicities
MDS-pCT #1
AML-pCT #2
AML-pCT #3
Severe (G≥3)
Grade of toxicity
4
3
2
1
0
CRS
ICANS
early ICAHT
late ICAHT
B
MDS-pCT #1
6000
4000
2000
0
20
15
10
5
0
ng/mL
mg/dL
0
10
20
30
110
190
270
Days relative to CAR-T infusion
C
AML-pCT #2
1500
1000
500
0
15
10
5
0
ng/mL
mg/dL
0
10
20
30
110
190
270
Days relative to CAR-T infusion
D
AML-pCT #3
1500
1000
500
0
6
4
2
0
ng/mL
mg/dL
0
10
20
30
110
190
270
Days relative to CAR-T infusion
Ferritin
CRP
